# Treatment with Polyethylene Glycol–Conjugated Fungal d-Amino Acid Oxidase Reduces Lung Inflammation in a Mouse Model of Chronic Granulomatous Disease

**DOI:** 10.1007/s10753-022-01650-z

**Published:** 2022-02-24

**Authors:** Hiroyuki Nunoi, Peiyu Xie, Hideaki Nakamura, Yasuaki Aratani, Jun Fang, Toyoki Nishimura, Hiroaki Kataoka, Hiroshi Maeda, Makoto Matsukura

**Affiliations:** 1 Division of Pediatrics, Faculty of Medicine, University of Miyazaki, 5200 Kihara, Kiyotake-cho, Miyazaki City, 889-1692 Miyazaki, Japan; 2 Aisenkai Nichinan Hospital, 3649-2 Kazeta, Nichinan City, Miyazaki 887-0034 Japan; 3 Laboratory of Clinical Pharmacology and Therapeutics, Faculty of Pharmaceutical Sciences, Sojo University, 4-22-1 Ikeda, Nishi-ku, Kumamoto City, 860-0082 Japan; 4 Laboratory of Microbiology & Oncology, Faculty of Pharmaceutical Sciences, Sojo University, 4-22-1 Ikeda, Nishi-ku, Kumamoto City, 860-0082 Japan; 5 Graduate School of Nanobioscience, Yokohama City University, 22-2 Seto, Kanazawa Ward, Yokohama, Kanagawa 236-0027 Japan; 6 Laboratory of Microbiology, Faculty of Pharmacological Sciences, Sojo University, 4-22-1 Ikeda, Kumamoto Nishi-ku Kumamoto City, 860-0082 Japan; 7 Department of Pathology, University of Miyazaki, 5200 Kihara, Kiyotake-cho, Miyazaki-shi Miyazaki, 889-1692 Japan; 8 BioDynamics Research Foundation, Bldg. 3F, 1-24-6 Kuwamizu, Chuo-ku, Kumamoto, 862- 0954 Japan

**Keywords:** nCA-induced lung inflammation, CGD mice, PEG-d-amino acid oxidase (PEG-fDAO), enzyme replacement therapy.

## Abstract

Chronic granulomatous disease (CGD) is a primary immunodeficiency wherein phagocytes are unable to produce reactive oxygen species (ROS) owing to a defect in the nicotinamide adenine dinucleotide phosphate oxidase (NADPH) complex. Patients with CGD experience bacterial and fungal infections and excessive inflammatory disorders. Bone marrow transplantation and gene therapy are theoretically curative; however, residual pathogenic components cause inflammation and/or organic damage in patients. Moreover, antibiotic treatments may not help in preventing excessive inflammation due to the residual presence of fungal cell wall β-glucan. Thus, better treatment strategies against CGD are urgently required. Polyethylene glycol–conjugated recombinant porcine d-amino acid oxidase (PEG-pDAO) supplies ROS to defective NADPH oxidase in neutrophils of patients with CGD, following which the neutrophils regain bactericidal activity *in vitro*. In this study, we employed an *in vivo* nonviable *Candida albicans* (nCA)–induced lung inflammation model of gp91-phox knockout CGD mice and supplied novel PEG conjugates of *Fusarium* spp. d-amino acid oxidase (PEG-fDAO), as it exhibits higher enzyme activity than PEG-pDAO. The body weight, lung weight, and lung pathology were evaluated using three experimental strategies with the *in vivo* lung inflammation model to test the efficacy of the ROS-generating enzyme replacement therapy with PEG-fDAO. The lung weight and pathological findings suggest the condition was ameliorated by administration PEG-fDAO, followed by intraperitoneal injection of d-phenylalanine or d-proline. Although a more precise protocol is essential, these data reveal the targeted delivery of PEG-fDAO to the nCA-induced inflammation site and show that PEG-fDAO can be used to treat inflammation in CGD *in vivo*.

**Supplementary Information:**

The online version contains supplementary material available at 10.1007/s10753-022-01650-z.

## INTRODUCTION

Chronic granulomatous disease (CGD) is a primary immunodeficiency characterized by the inability of phagocytes to produce reactive oxygen species (ROS) owing to a defect in the nicotinamide adenine dinucleotide phosphate (NADPH) oxidase complex. The average worldwide birth prevalence of CGD is estimated between 1/100,000 and 1/217,000. Clinically, most patients with CGD experience bacterial and fungal infections in their childhood and have excessive inflammatory disorders, such as CGD-associated bowel inflammation and sterile granulomas. ROS generation is crucial for inhibiting the growth of microbes ingested by phagocytes [1] and for inflammasome signaling processes, such as caspase-1 activation of cytokine production [2–4], the Keap1-Nrf2 pathway for antioxidative stress [5], and efferocytosis through phosphatidylserine and its receptor [6].

Anti-inflammatory cytokine therapies (using anti-interleukin [IL]-1 antibody) alleviate excessive cytokine production in an *in vivo* CGD mouse model and demonstrate efficacy in clinical studies on severe colitis [4]. In addition, treatment with a peroxisome proliferator-activated receptor gamma (PPARγ) agonist (pioglitazone) increases mitochondrial ROS production and partially restores host defense in the CGD mouse model [6]. Bone marrow transplantation or gene therapy with a lentiviral vector and a myeloid-specific promoter have been proposed as curative [7]. However, residual pathogenic components, live or dead, cause vital reactions, such as inflammation and/or organic damage, in patients with CGD. Moreover, antibiotic treatments are used as pathogen-specific remedies for pathogen removal. Potent pathogen-specific antibiotic or antifungal treatment can kill pathogens; however, they may not prevent excessive inflammation that can persist owing to the presence of residual materials, such as fungal cell wall β-glucan, derived from the killed pathogens. Furthermore, persistent chronic inflammation in patients with CGD leads to impaired hematopoietic stem cell function [8]. Therefore, there is an urgent need for better treatment strategies.

In this study, we aimed to develop a novel enzyme replacement therapy with d-amino acid oxidase (DAO) to supply H_2_O_2_ *in vivo*. DAO is a flavoenzyme that selectively catalyzes the oxidative deamination of d-amino acids, thereby generating the corresponding amino acid and H_2_O_2_. Furthermore, DAO has been shown to restore the bactericidal activity of ROS-deficient neutrophils by supplying H_2_O_2_ *in vitro* [9]. There are advantages in using fungal DAO, including ready availability and a more potent selective activity than that of mouse or human DAO [10, 11]. Moreover, the drawback of immunogenicity owing to the fungal origin may be overcome by PEGylation or chemical modification of the enzyme, which nullifies the immunogenicity and confers the PEGylated enzyme a much longer plasma half-life [10]. Such macromolecules tend to accumulate more selectively in inflamed tissues as well as in cancer tissues. This phenomenon is known as the enhanced permeability and retention effect [11].

Here, we characterized a novel polyethylene glycol–conjugated *Fusarium* spp. DAO (PEG-fDAO) and evaluated its *in vivo* anti-inflammatory activity in a mouse model in which lung inflammation is induced using nonviable *Candida albicans* (nCA) in gp91-phox knockout CGD mice. Lack of ROS production results in high levels of proinflammatory mediators (IL-1b, tumor necrosis factor-α, and keratinocyte chemoattractant) via the inflammasome activation system (dectin-1 receptor) in neutrophils and macrophages [3]. This model mimics the fact that viable fungi are not always a prerequisite for developing inflammation in the CGD mice [12]. Using this model, we propose that oxidase replacement therapy with the novel PEG-fDAO may have applications in treating inflammation in CGD *in vivo*.

## MATERIALS AND METHODS

### Reagents

DAO isolated from *Fusarium* spp. (fDAO) was provided by Ikeda Food Research Co., Ltd. (Hiroshima, Japan). Succinimide-PEG (ME-020CS) was purchased from Nippon Oil & Fat Co., Ltd. (Tokyo, Japan). Bovine serum albumin (BSA), rhodamine B isothiocyanate, and ethylenediaminetetraacetic acid (EDTA) were purchased from Wako Pure Chemical Industries, Ltd. (Osaka, Japan). d-alanine, d-proline, d-phenylalanine, d-proline, d-methionine, and d-leucine were purchased from Peptide Institute Inc. (Osaka, Japan). Phenylmethylsulphonyl fluoride (PMSF) and leupeptin were purchased from Merck KGaA (Darmstadt, Germany). Eosin and Mayer’s hematoxylin solution were purchased from Sakura Finetek Japan Co., Ltd (Tokyo, Japan). All other reagents were of reagent grade and used without further purification.

### Animals

CGD mice (gp91-phox knockout) obtained from Dr. Mary C. Dinauer [13] were backcrossed at least 12 times with C57BL/6 mice to ensure similar genetic backgrounds. Experiments on 8–12-week-old C57BL/6 mice were performed according to the guidelines of the Laboratory Protocol of Animal Handling, Sojo University Faculty of Pharmaceutical Sciences. All animals were housed under specific pathogen-free conditions. This study was approved by the Ethics Review Board of the Faculty of Pharmaceutical Sciences (Permission number: 2017-P-027).

### Preparation of nCA

nCA was provided by Dr. Ohno [14]. Briefly, a C-limiting medium was used to grow *C. albicans* and which was cultured at 27 °C with aeration. Viable cells were collected via centrifugation, killed with ethanol, and dried with acetone. The resulting nCA was suspended in phosphate-buffered saline (PBS).

### Preparation of PEG-fDAO

PEGylation of fDAO was performed as previously described [15]. In brief, succinimide-activated PEG was added at a threefold molar excess of PEG to the fDAO solution (2.0 mg/mL protein in 0.1 M sodium bicarbonate) to free the amino groups in fDAO and allowed to react for 1 h at 4 °C. The reaction mixture containing PEG-fDAO was purified to remove free PEG and other low molecular weight reactants by ultrafiltration with a YM-10 membrane (Merck, Darmstadt, Germany) using 0.1 M sodium bicarbonate aqueous solution. Thereafter, the purified PEG-fDAO was stored at −80 °C until further use.

### Evaluation of fDAO and PEG-fDA Enzymatic Activity

The enzymatic activity of fDAO and PEG-fDAO was determined via a horseradish peroxidase-coupled colorimetric assay with *o*-dianisidine as the substrate. In this assay, the substrate was reduced, and the color developed revealed maximal absorption of 460 nm. d-Alanine was used as the substrate for fDAO at a final concentration of 10 mM. The enzymatic reaction was performed at 25 °C in 0.1 M Tris–HCl buffer (pH 8.2), where 1 U of fDAO activity was defined as the rate of formation of 1 μmol of H_2_O_2_ per minute. The maximal rate of activity (Vmax) and Km for each d-amino acid were calculated by curve fitting of the nonlinear plot of reaction rate *versus* substrate concentration using the gnuplot software.

### Analysis of *In Vivo* Pharmacokinetics of fDAO and PEG-fDAO Using Plasma

fDAO or PEG-fDAO (20 U/mL, 0.1 mL/mouse, *n* = 3) was injected intravenously into C57BL/6 (BALB/cAjcl) mice for *in vivo* pharmacokinetics analysis. Blood was withdrawn from the medial canthus of the eye using a microhematocrit at 0.5, 4, 24, 48, and 72 h after fDAO or PEG-fDAO administration. Thereafter, each blood sample was centrifuged, and the plasma was obtained in an ice-cold buffer (100 mM Tris–HCl, pH 8.0) containing a mixture of protease inhibitors (1 mM PMSF, 10 μg/mL leupeptin, and 2.5 mM EDTA). The DAO activity in the plasma was determined based on the formation of pyruvic acid during the reaction between d-alanine and DAO, as previously described [15].

### Induction of Lung Inflammation via nCA Aspiration

Mice were anesthetized intraperitoneally with 200 mg/kg of 2,2,2-tribromoethanol (Sigma-Aldrich) injection, as described by Dr. Aratani [12]. The CGD mice were subjected to intranasal administration of 10^7^ nCA cells in a volume of 30 μL PBS, and the control mice were administered 30 μL of PBS alone [12]. Short, medium, and long treatment schedules were explored as the time course of *in vivo* production of H_2_O_2_ induced by PEG-fDAO with d-amino acids in the nCA-induced lung inflammation model was unknown.

### Experiment-1

Mice were divided into two groups. The first group was administered nCA intranasally as a positive control (*n* = 5). The second group was administered nCA intranasally on day 0 of the experiment, and PEG-fDAO (10 U/mL, 0.1 mL/mouse) was injected through the tail vein on day 2, followed by intraperitoneal administration of d-phenylalanine (0.1 M, 0.5 mL) on days 3, 4, and 5 (*n* = 5). The mice were sacrificed on day 8.

### Experiment-2

Mice were divided into three groups. The first group was administered PBS intranasally as a negative control (*n* = 3). The second group was administered nCA intranasally as a positive control (*n* = 3). The third group was administered nCA on day 0 and injected with PEG-fDAO (10 U/mL, 0.1 mL/mouse) (*n* = 3) through the tail vein on day 4, followed by intraperitoneal injection of d-phenylalanine (0.1 M, 0.5 mL/mouse) on days 5, 6, and 7 instead of d-proline. Rhodamine-labeled bovine serum albumin (rhodamine-BSA) (10 mg/kg) was injected 1 day before sacrifice on the 14th day. Fluorescence imaging of the excised lungs was performed using the IVIS Lumina XR (excitation: 555–585 emission: 695–770 nm; PerkinElmer Japan Co., Ltd., Kanagawa, Japan).

### Experiment-3

Mice were divided into three groups. The first group was administered PBS intranasally as a negative control (*n* = 3). The second group was administered nCA intranasally as a positive control (*n* = 3). The third group was administered nCA on day 0 and injected with PEG-fDAO (10 U/mL, 0.1 mL/mouse) (*n* = 3) on day 9, followed by intraperitoneal injection of d-proline (1 M, 0.5 mL/mouse) on days 10, 11, and 12. Rhodamine-BSA (10 mg/kg) was injected one day before sacrifice on day 21. Fluorescence imaging of the excised lungs was performed using an IVIS Lumina XR (PerkinElmer Japan Co., Ltd.).

### Quantification of Fluorescence Imaging of Rhodamine-BSA

The photon intensity images, after subtracting the photo intensity of the red channel from the blue channel, were analyzed by ImageJ and are illustrated in Supplement Figs. 2 and 3. The photon volumes of each sample were shown for quantitative analysis, which were calculated by multiplying the photon intensity and spot numbers by ImageJ.

### Analysis of Lung Pathology

Mice were sacrificed on day 8 in Experiment-1 (Exp-1), day 14 in Experiment-2 (Exp-2), or day 21 in Experiment-3 (Exp-3) after nCA administration. The lungs were removed and fixed in 10% buffered formalin. Subsequently, the tissues were fixed overnight, dehydrated in graded ethanol solutions, embedded in paraffin, sectioned at 2 μm thickness, and stained with hematoxylin and eosin using standard protocols; images were observed under a light microscope (OLYMPUS BX51 with cellSens imaging software).

As the eosinophilic filaments were presumed to be undigested remnants of nCA in the absence of ROS production by host phagocytes, all eosinophilic filament-phagocytized cells were enumerated in 10 fields with × 400 magnification for each sample to quantify the severity of lung inflammation. The number of phagocytized cells was analyzed using quartile statistics, shown in Supplement Fig. 4.

### Statistical Analysis

Statistical analysis of the data was performed using a two-tailed unpaired Welch *t*-test. Differences were considered statistically significant at *p* < 0.05. In Supplement Fig. 4, the quartile statistics was performed.

## RESULTS

### Physicochemical Characteristics of fDAO and PEG-fDAO

The characteristics of PEG-fDAO are summarized as follows: The reaction between fDAO and succinimide-PEG resulted in PEG conjugates on fDAO with a chain number of five, which was determined by quantifying the primary amine. The fDAO formed a tetramer (165 kDa) in the physiological solution used for testing, and the molecular weight of PEG-fDAO was calculated to be 206 kDa. An increase in the molecular size of fDAO by PEGylation was further confirmed by SDS-PAGE and size-exclusion chromatography [15]. Enzyme-specific activity of fDAO was 26 ± 1.3 units/mg protein, and it remained unaltered by PEGylation (PEG-fDAO: 26 ± 0.53 units/mg protein). The enzyme-specific activity of PEG-fDAO was 3.6-fold higher than that of previously reported PEG-pDAO (7.3 units/mg protein), indicating that PEG-fDAO generates H_2_O_2_ more efficiently than does PEG-pDAO. The Vmax and Km values of fDAO for each d-type amino acid were similar to those of PEG-pDAO. Vmax and Km values of fDAO and PEG-fDAO toward neutral d-amino acids revealed similar trends at the optimal pH of 8.2, and at an inflammatory environmental pH of 6.5 (Supplement Table 1). Furthermore, the enzymatic activity of PEG-fDAO at pH 6.5 was lower than that at pH 8.2, for all substrates tested. Similar substrate specificity was observed at pH 6.5 and 8.2.

Among the d-amino acids tested, d-phenylalanine exhibited the highest Vmax (Vmax = 18.6 μmol H_2_O_2_/min at pH 6.5) and was active even at low substrate concentrations (Km = 0.22 mM at pH 6.5). d-Proline was used in Exp-3 to compare the *in vitro* bactericidal activity with the *in vivo* anti-inflammatory activity (Vmax = 6.4, Km = 5.2 at pH 6.5). In Exp-1 and Exp-2, d-phenylalanine was selected as the substrate to confirm PEG-fDAO activity *in vivo*.

### *In Vivo* Pharmacokinetics of fDAO and PEG-fDAO Using Plasma

**Fig. 1:**
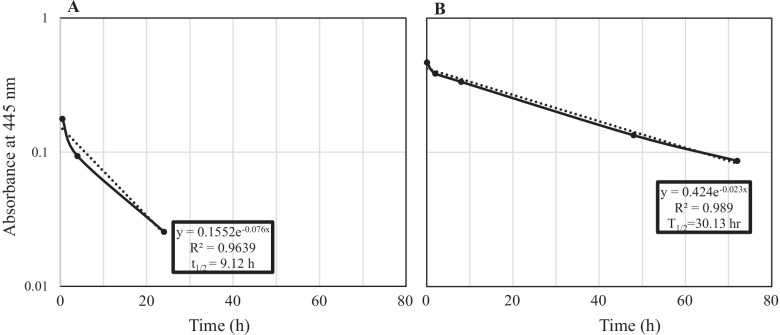
*In vivo* pharmacokinetics of fDAO and PEG-fDAO by enzymatic activities using plasma. The *y*-axis depicts the log of the absorbance at 445 nm, which reflects the production of pyruvic acid from d-alanine by d-amino acid oxidase. In this figure, two typical sets of exponential curve fitting analysis along with the mathematical formula are presented for fDAO (**A**) and PEG-fDAO (**B**) (Microsoft Excel^®^). The mathematical calculation for biological half-life (*x* = t1/2) is also depicted in the figure. R^2^ is *R* squared, which is the coefficient of determination that is predictable from the independent variable.

We employed a methodology to exclude the denatured DAO proteins with lost enzymatic activity to measure the enzymatic activity of DAO *in vivo* (Fig. 1). The apparent half-life (t1/2) of fDAO (*n* = 3) and PEG-fDAO (*n* = 7) was statistically 7.69 ± 1.36 h and 33.68 ± 3.10 h, respectively. A *t*-test with unpaired samples revealed a *p*-value of 0.000000415. Since the t1/2 of PEG-fDAO revealed a longer period, and lethal toxicity [15] of PEG-fDAO remained in the plasma, an intraperitoneal injection of d-amino acid was administered late on three consecutive days, instead of after a short lag time (7 and 20 h) after PEG-fDAO administration.

### *In Vivo* Experimental Findings

**Fig. 2:**
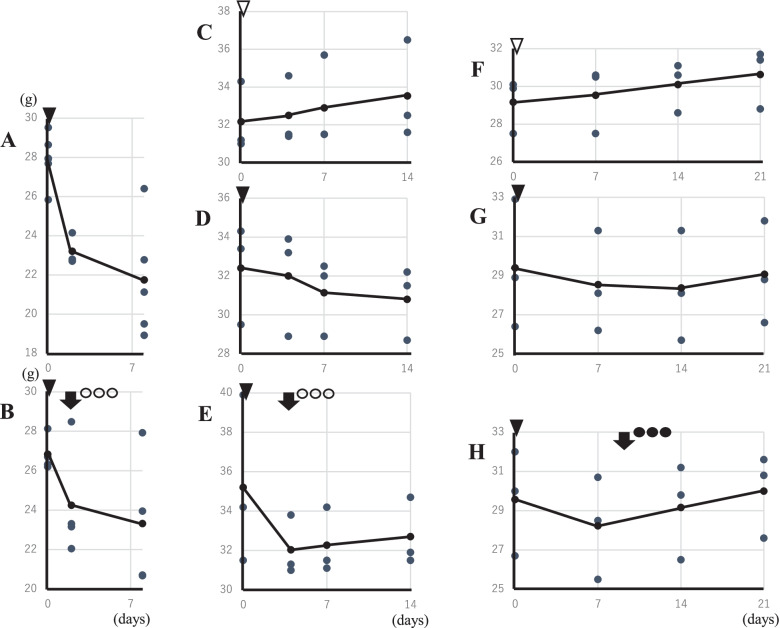
Changes in body weight in each experiment. CGD mice were intranasally administered 10^7^ nCA cells (black down-pointing triangle) in a volume of 30 μL of PBS, and control mice were administered 30 μL of PBS alone (white down-pointing triangle). PEG-fDAO (black downward arrow) was intravenously administered on the second day (**B**), fourth day (**E**), and ninth day (**H**), followed by intraperitoneal administration of d-phenylalanine (white circle) or d-proline (black circle). The small dots indicate the body weight (*y*-axis) in the time course (*x*-axis) of each experiment. The solid line indicates the mean body weight. Since the age of the mice differed during commencement of each experiment, the average body weight also differed across the experiments.

**Fig. 3:**
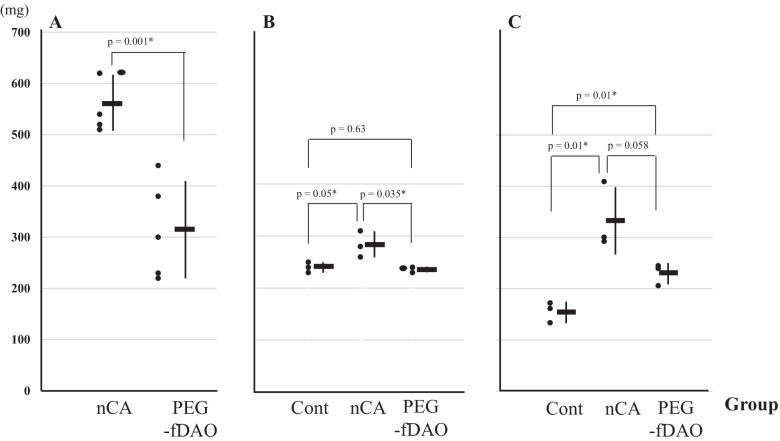
Change in the lung weight in each experiment. The small dots (black circle) depict the lung weight in Exp-1/-2/-3 (**A**/**B**/**C**). Solid bars (**–**) and the vertical bars indicate mean and ± 2 SD of the lung weight (mg) in control, nCA, and PEG-fDAO groups. An asterisk (*) indicates the significant difference between the groups in each experiment.

**Fig. 4:**
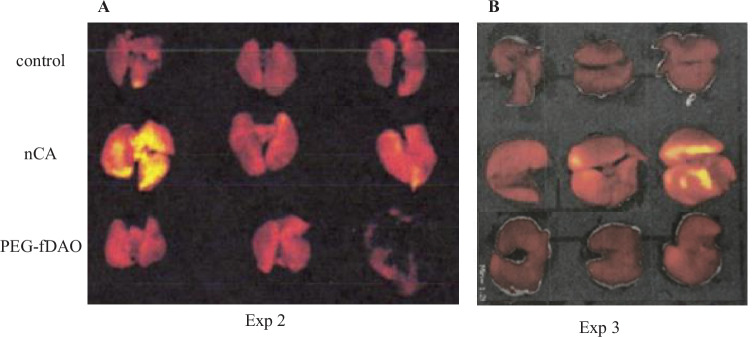
Fluorescence imaging of the lung tissues during each experiment. The lung tissues of mice from the control, nCA, and PEG-fDAO groups in Exp-2 (**A**) and Exp-3 (**B**) are depicted.

**Fig. 5:**
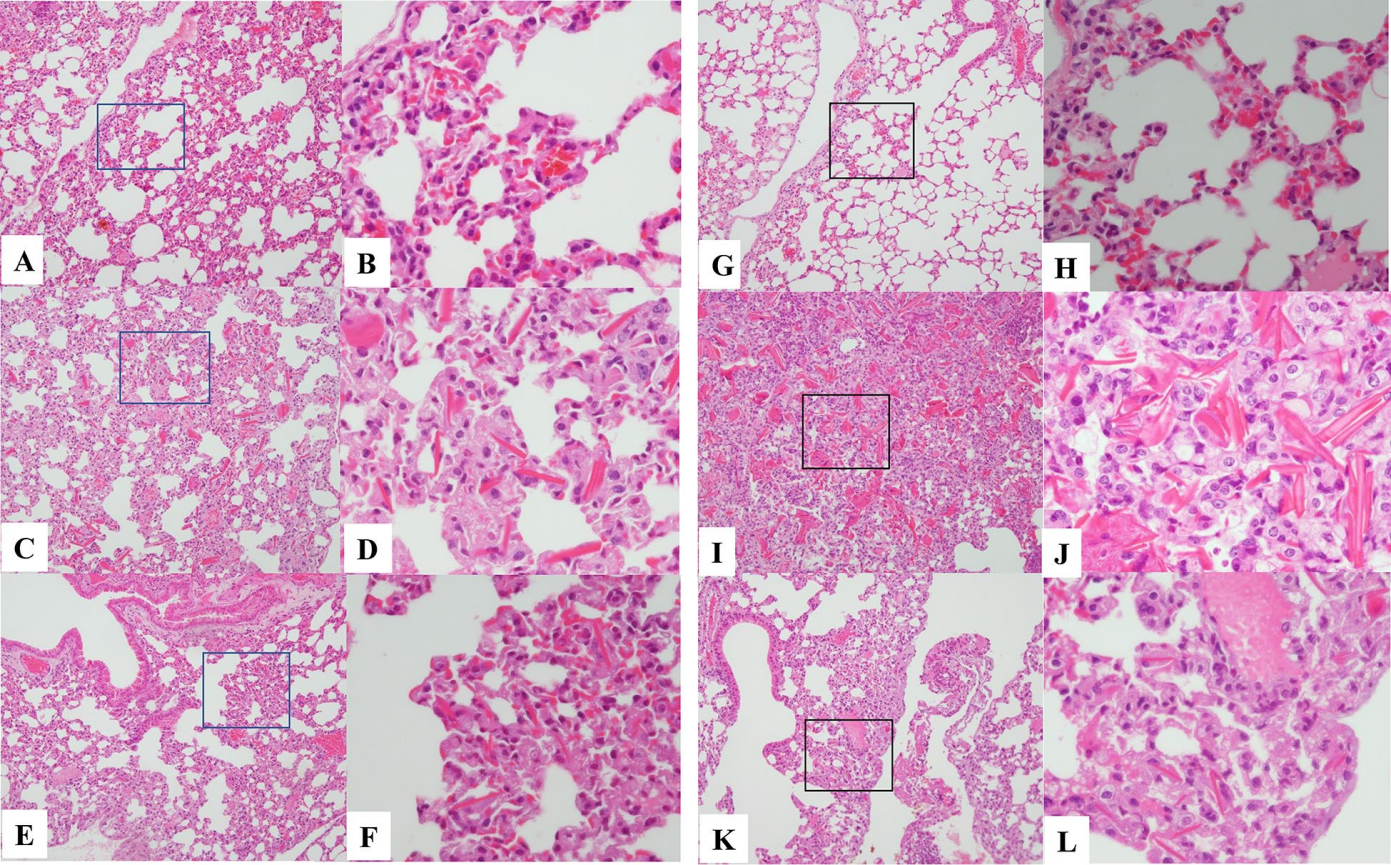
Pathological findings of the lung tissues in Exp-2 and Exp-3. Lung pathologies in **A**–**F** Exp-2 and **G**–**L** Exp-3 are shown, where **A**/**B** and **G**/**H**, **C**/**D** and **I**/**J**, and **E**/**F** and **K**/**L** indicate the control, nCA, and PEG-fDAO group, respectively. **A**/**C**/**E**/**G**/**I**/**K** and **B**/**D**/**F**/**H**/**J**/**L** indicate the original magnifications of × 100 and × 400, respectively. The boxed areas are amplified in the right panels. The nCA group revealed pneumonia with numerous eosinophilic filament-phagocytized cells in **C**/**D** Exp-2 and **I**/**J** Exp-3. In addition, slight granulomatous appearance indicating a mixed accumulation of neutrophils, macrophages, and lymphoid cells were noted in **I**/**J** Exp-3. The inflammatory changes were milder in the **E**/**F**/**K**/**L** PEG-fDAO group than in the **C**/**D**/**I**/**J** nCA group, indicating thin eosinophilic filament-phagocytized cells in both Exp-2 and Exp-3.

1. Changes in body and lung weight
  In Exp-1 with an 8-day schedule, a marked mean body weight loss was noticed in the nCA group (−6.18 ± 2.28 g) compared with the PEG-fDAO experimental group (−4.14 ± 2.74 g); however, the difference was not significant (*p* = 0.236, *n* = 5; Fig. 2A, B). The mean lung weight in the nCA group (0.562 ± 0.04 g) was significantly higher than that in the PEG-fDAO experimental group (0.314 ± 0.095 g) (*p* = 0.001, *n* = 5; Fig. 3A). Images of the excised lungs are shown in Supplement Fig. 1.
  In Exp-2 with a 14-day schedule and d-phenylalanine substrate, the mean body weight loss in the nCA group (−1.6 ± 0.7 g) was lower than that in the PEG-fDAO group (−2.5 ± 2.8 g) (*p* = 0.618, *n* = 3; Fig. 2D, E). Moreover, the mean lung weight in the nCA group (0.283 ± 0.025 g) was significantly higher than that in the PEG-fDAO group (0.236 ± 0.005 g) (*p* = 0.035, *n* = 3; Fig. 3B). Furthermore, the mean lung weight in the control group was 0.24 ± 0.01 g, which was almost similar to that in the PEG-fDAO group (*p* = 0.64, *n* = 3; Fig. 3B).
  In Exp-3 with a 21-day schedule, no marked difference was observed in the mean body weight loss in the nCA group (−0.33 ± 0.68 g) than that in the PEG-fDAO group (−0.17 ± 0.87 g) (*p* = 0.66, *n* = 3; Fig. 2G, F). The mean lung weight in the nCA group (0.334 ± 0.065 g) was higher than that in the PEG-fDAO group (0.230 ± 0.021 g); however, no significant difference was observed (*p* = 0.058, *n* = 3; Fig. 3C). In the control group, the mean lung weight was 0.156 ± 0.020 g.
2. Rhodamine-BSA analysis
  To highlight inflammatory regions in the lung, rhodamine-BSA was intravenously injected, and the fluorescence intensity of accumulated rhodamine-BSA in the lung (Fig. 4) was analyzed using Image J software.
  In Exp-2 and Exp-3 (Fig. 4A, B), rhodamine-labeled fluorescence intensity in the excised lungs of mice from the nCA group appeared brighter than that in the PEG-fDAO and control groups. We quantitatively analyzed the images (Fig. 4). Subtracted photon intensity profiles of the red channel from the blue channel analyzed by ImageJ are illustrated in Supplement Figs. 2 and 3. The distribution of the subtracted photon intensity was analyzed, and the photon volumes of each sample were calculated by multiplying the photon intensity with the spot numbers. As depicted in Supplement Fig. 4B, the photon volumes of the nCA group were significantly higher (*p* = 0.014) than those of the PEG-fDAO group; however, as shown in Supplement Fig. 4A, these volumes of the nCA group were apparently higher but not significant (*p* = 0.062).
3. Pathological analysis
  The pathological findings differed in the nCA groups (Fig. 5C/D and I/J), revealing pneumonia with accumulation of eosinophilic filament-phagocytized cells, accompanied by granulomatous accumulation of histiocytic cells and lymphoid cells; however, these findings appeared milder in the PEG-fDAO group (Fig. 5E/F and K/J). Moreover, a small amount of granuloma formation with giant cells was observed in the PEG-fDAO group. The larger crystalized filaments were prominent in the nCA group, whereas the thinner filaments were prominent in the PEG-fDAO group (Fig. 5D/J *vs.* F/L).

To quantify the pathological severity between the nCA and PEG-fDAO groups, we enumerated the eosinophilic filament-phagocytized cells in the 10 separate fields with × 400 magnification where the phagocytized cells were counted more than once. The results are shown in Supplement Fig. 4, where the number of phagocytized cells in a field is depicted (o) and was analyzed by quartile statistics. In Exp-2 and Exp-3, the number of phagocytized cells in the nCA group was out of the interquartile range (IQR; bar plot) of that in the PEG-fDAO group (Supplement Fig. 4A and B).

## DISCUSSION

According to studies on *Aspergillus* hyphae-induced [16] and sterile nCA-induced [12] lung inflammation models, the sterile fungal cell wall (branched (β-1,3) (β-1,6) glucan)-induced inflammation model with gp91^−/−^ CGD mice [17] and zymosan (the same fungal cell wall-derived product)-induced lung inflammation model with p47^−/−^ [18] and p91^−/−^ [19] CGD mice, the MAPK signaling pathway (ERK1/2 and NF-κB) involving Toll-like receptor 2, dectin-1 receptor, cytokines (IL-1b, tumor necrosis factor-α, IL-17, keratinocyte chemoattractant, and granulocyte colony-stimulating factor), and leukotriene B4 [19] was activated on the second day or earlier poststimulation. The net balance of cytokine and leukotriene B4 expression in the lungs of CGD mice in response to the fungal cell wall component is prone to be in a proinflammatory state, which may trigger continuous inflammatory response [16, 19].

Our pharmacokinetics analysis revealed that fDAO (165 kDa) was rapidly cleared from the circulation after intravenous infusion (t1/2 = 7.67 ± 1.36 h (*n* = 3)), but PEG-fDAO (206 kDa) was rather stable (t1/2 = 33.68 ± 3.10 h (*n* = 7)) in the present study (Fig. 1). In addition, Nakamura *et al.* reported that the high enzyme activity of PEG-fDAO in plasma limited repeated treatment owing to lethal toxicity [15]. Based on these pharmacokinetic and toxic effects of PEG-fDAO, to evaluate its anti-inflammatory effect, d-amino acid was intraperitoneally injected in 3 consecutive days instead of after a short lag time (7 and 20 h) after PEG-fDAO administration. The Km value of d-phenylalanine was approximately 24–40 times lesser than that of d-proline. Since the blood concentration of d-phenylalanine or d-proline was presumed to be 2–3 times higher than the Km value of both d-amino acids, the Vmax of PEG-fDAO for d-phenylalanine was approximately three times higher than that of d-proline (Supplement Table 1). Since d-phenylalanine has lower solubility than d-proline in aqueous media, bolus injectable d-phenylalanine was tenfold lower than d-proline (d-phenylalanine: 0.1, 0.5 mL/mouse (Exp-1 and Exp-2) and d-proline: 1 M, 0.5 mL/mouse (Exp-3). Although PEG-fDAO activity was higher toward d-phenylalanine than d-proline, a low dose of d-phenylalanine might result in a therapeutic efficacy comparable to that of d-proline in alleviating lung inflammation, more stable conditions and protocols are necessary to confirm the apparent effects of PEG-fDAO observed in this model and to develop clinical applications.

In the first experiment Exp-1, we administered PEG-fDAO on day 2, followed by an intraperitoneal injection of d-phenylalanine for 3 days. Endo *et al.* [12] reported that nCA-treated CGD mice revealed prominent airway accumulation of inflammatory cells at days 3 and 6 post-nCA administration. Similarly, the nCA-treated CGD mice in Exp-1 presented with a prominent loss in body weight and increase in lung weight. In contrast, the nCA-treated CGD mice injected with PEG-fDAO and d-phenylalanine revealed less decrease in body weight (*p* = 0.035) and increase in lung weight (*p* = 0.001) (Figs. 2A/B and A). These differences were also apparent from a physiological point of view.

Moreover, to determine the effect of PEG-fDAO over a longer period, comparable to that used in the *Aspergillus* hyphae-induced lung inflammation model [16], PEG-fDAO was administered on day 4 in Exp-2 with a 14-day schedule, and on day 9 in Exp-3 with a 21-day schedule.

In Exp-2, the loss of body weight in the nCA group was similar to that in the PEG-fDAO group (*p* = 0.62; Fig. 2D/E); however, the increase in lung weight was significantly higher in the nCA group (*p* = 0.035) than that in the PEG-fDAO group (Fig. 3B).

In Exp-3, the loss of body weight in the nCA group was higher than that in the PEG-fDAO group, but the difference was not significant (*p* = 0.66) (Fig. 2G/H). The gain in lung weight in the nCA group was not significantly higher (*p* = 0.058) than that in the PEG-fDAO group (Fig. 3C).

In Exp-2 and Exp-3 (Fig. 4A, B), rhodamine-labeled fluorescence intensity in the excised lungs of mice from the nCA group appeared brighter than that in the PEG-fDAO and control groups. We quantitatively analyzed the images (Fig. 4) by ImageJ. The photon volume of the nCA group was apparently higher (*p* = 0.064) in Exp-2, and significant (*p* = 0.014) in Exp-3 than that in the PEG-fDAO group (Supplement Fig. 3).

Granulomatous formations were not apparent in Exp-2, but a few macrophages accumulated around the eosinophilic *Candida* bodies in the pathological lung specimens from mice in the nCA group (Fig. 5C/D). On the contrary, several granulomatous formations were observed in Exp-3 in the pathological lung specimens in the nCA group (Fig. 5I/J), but only a few were observed in the PEG-fDAO group (Fig. 5K/L). These results are in accordance with a previous study by Dinauer [16], in which *Aspergillus fumigatus* hyphae were administered intratracheally to gp91^−/−^ mice for 21 days.

The larger crystallized filaments were prominent in the nCA group (Fig. 5C/D and I/J), and thinner filaments were observed in the PEG-fDAO group (Fig. 5 E/F and K/L). When the pathological changes in the lung were quantified by enumerating the eosinophilic filament-phagocytized cells in the 10 fields with × 400 amplification in each sample, the number of the cells in the fields of the nCA group were out of the interquartile range (IQR) of that in the PEG-fDAO group by the quartile statistics (Supplement Fig. 4A and B).

Although recovery of body weight was not evident (*p* = 0.035 in Exp-1, *p* = 0.62 in Exp-2, and *p* = 0.66 in Exp-3), lung inflammation in the nCA group was improved by the administration of PEG-fDAO and d-amino acid (Fig. 5C/D to E/F and I/J to KL) as indicated by lung weight (*p* = 0.001 in Exp-1, *p* = 0.035 in Exp-2, and *p* = 0.058 in Exp-3), accumulation of rhodamine-BSA (*p* = 0.062 in Exp-2 and *p* = 0.014 in Exp-3), and pathological changes (out of interquartile range). Therefore, in this series of experiments, we concluded that PEG-fDAO administration followed by d-amino acid injection was useful for treating nCA-induced lung inflammation.

Considering the mechanism of the nCA-induced pneumonia model, H_2_O_2_ appears to be involved in several inflammatory processes. As Endo *et al.* [12] reported in their 3-day pneumonia model, a low number of MAPKs in CGD mice may lead to prolonged phosphorylation of ERK1/2. Segal *et al.* [18] revealed impaired Nrf2 activity and increased NF-κB activation in zymosan-treated mononuclear cells from patients with X-linked CGD. Furthermore, numerous cytokines are involved in granuloma formation in giant cells [20], wherein H_2_O_2_ is involved. In lieu of neutrophil-derived ROS, the targeted delivery of PEG-fDAO could also restore the anti-inflammatory responses by supplying H_2_O_2_ to the site of inflammation. In similar fashion, Setoguchi *et al.* [21] reported a therapeutic value in controlling nitric oxide and ROS for treatment of pulmonary granulomas.

## CONCLUSIONS

We prepared the novel PEG-fDAO, characterized its enzymatic properties, and evaluated its anti-inflammatory effect on gp91-phox knockout CGD mice. The features of PEG-fDAO were comparable to those of pDAO in terms of Vmax and substrate specificity. PEG-fDAO was more stable and active than PEG-pDAO. Moreover, in our experiments with 3 different protocols, PEG-fDAO exhibited anti-inflammatory effects in CGD mice with nCA-induced lung inflammation *in vivo* and thus may be a useful candidate for enzyme replacement therapy. A protocol with an optimized regimen (either an administration schedule or a repetitive administration trial of PEG-fDAO) is required to confirm the apparent anti-inflammatory effects of PEG-fDAO.

## Supplementary Information

Below is the link to the electronic supplementary material.

Supplementary file1 (PPTX 2473 KB)

Supplementary file2 (PPTX 8416 KB)

## Data Availability

For original data and materials, please contact hnunoi@med.miyazaki-u.ac.jp.
