# Supplementary material for: Treatment with Polyethylene Glycol–Conjugated Fungal d-Amino Acid Oxidase Reduces Lung Inflammation in a Mouse Model of Chronic Granulomatous Disease
Source: Inflammation. 2022 Feb 24;45(4):1668–79. doi: 10.1007/s10753-022-01650-z (PMC9197883; doi:10.1007/s10753-022-01650-z)
Supplement: Supplementary file 1 — Supplementary file1 (PPTX 2473 KB) [file 10753_2022_1650_MOESM1_ESM.pptx]

## Slide 1
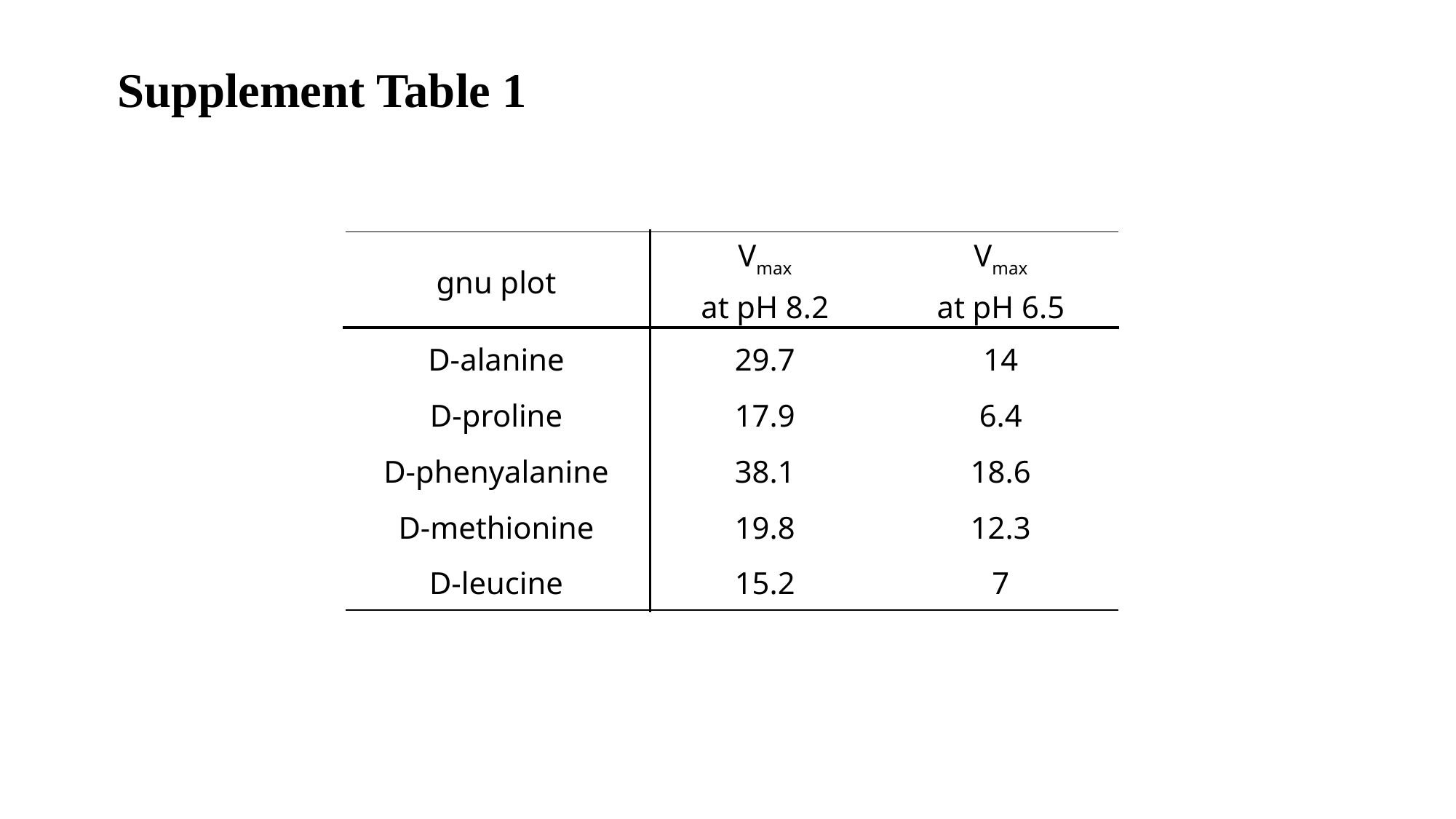

# Supplement Table 1
| gnu plot | Vmax | Vmax |
| --- | --- | --- |
| | at pH 8.2 | at pH 6.5 |
| D-alanine | 29.7 | 14 |
| D-proline | 17.9 | 6.4 |
| D-phenyalanine | 38.1 | 18.6 |
| D-methionine | 19.8 | 12.3 |
| D-leucine | 15.2 | 7 |

## Slide 2
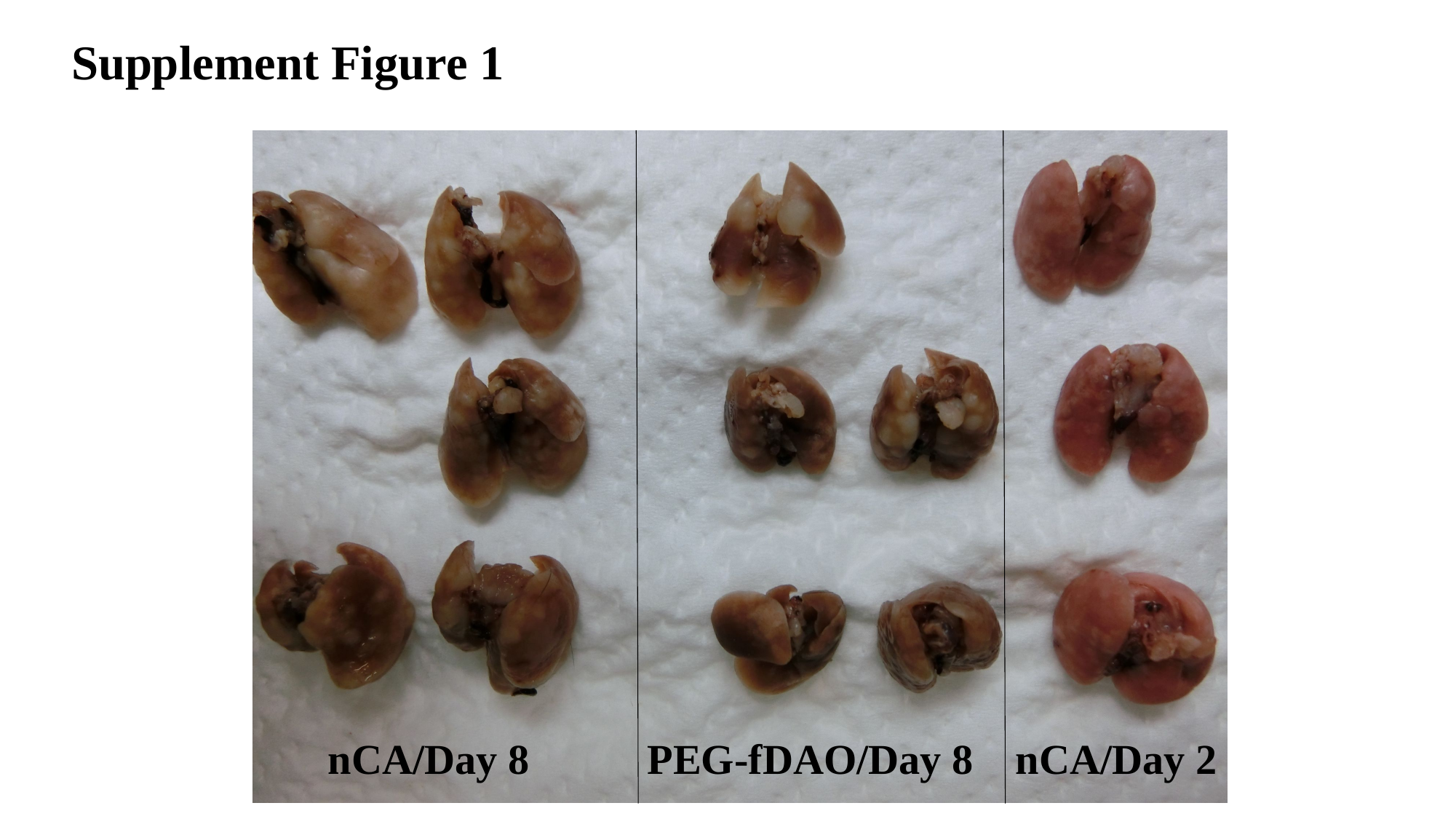

Supplement Figure 1
nCA/Day 8
PEG-fDAO/Day 8
nCA/Day 2

## Slide 3
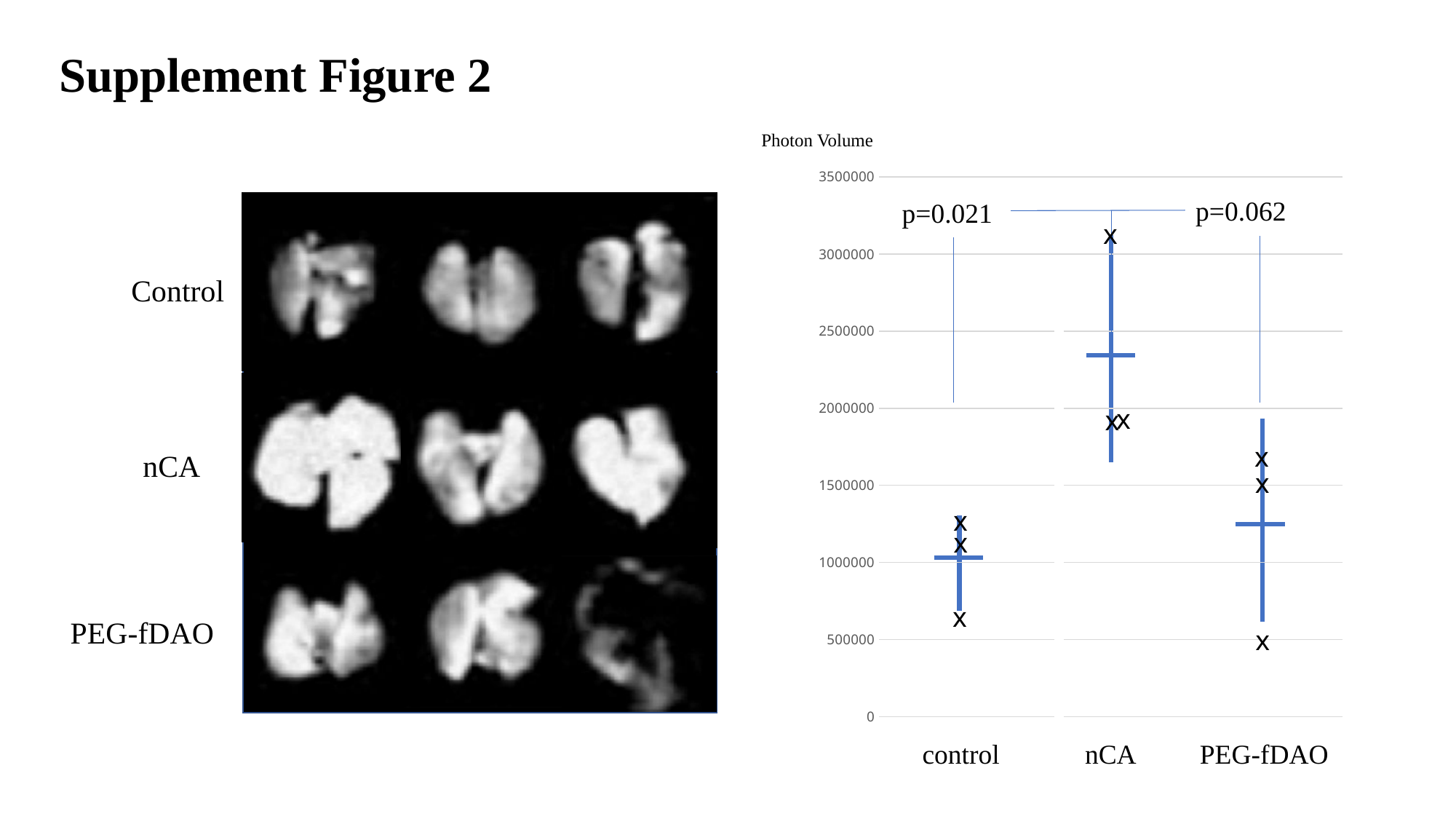

Supplement Figure 2
Photon Volume
[unsupported chart]
p=0.062
p=0.021
x
Control
x
x
x
nCA
x
x
x
x
PEG-fDAO
x
control
nCA
PEG-fDAO

## Slide 4
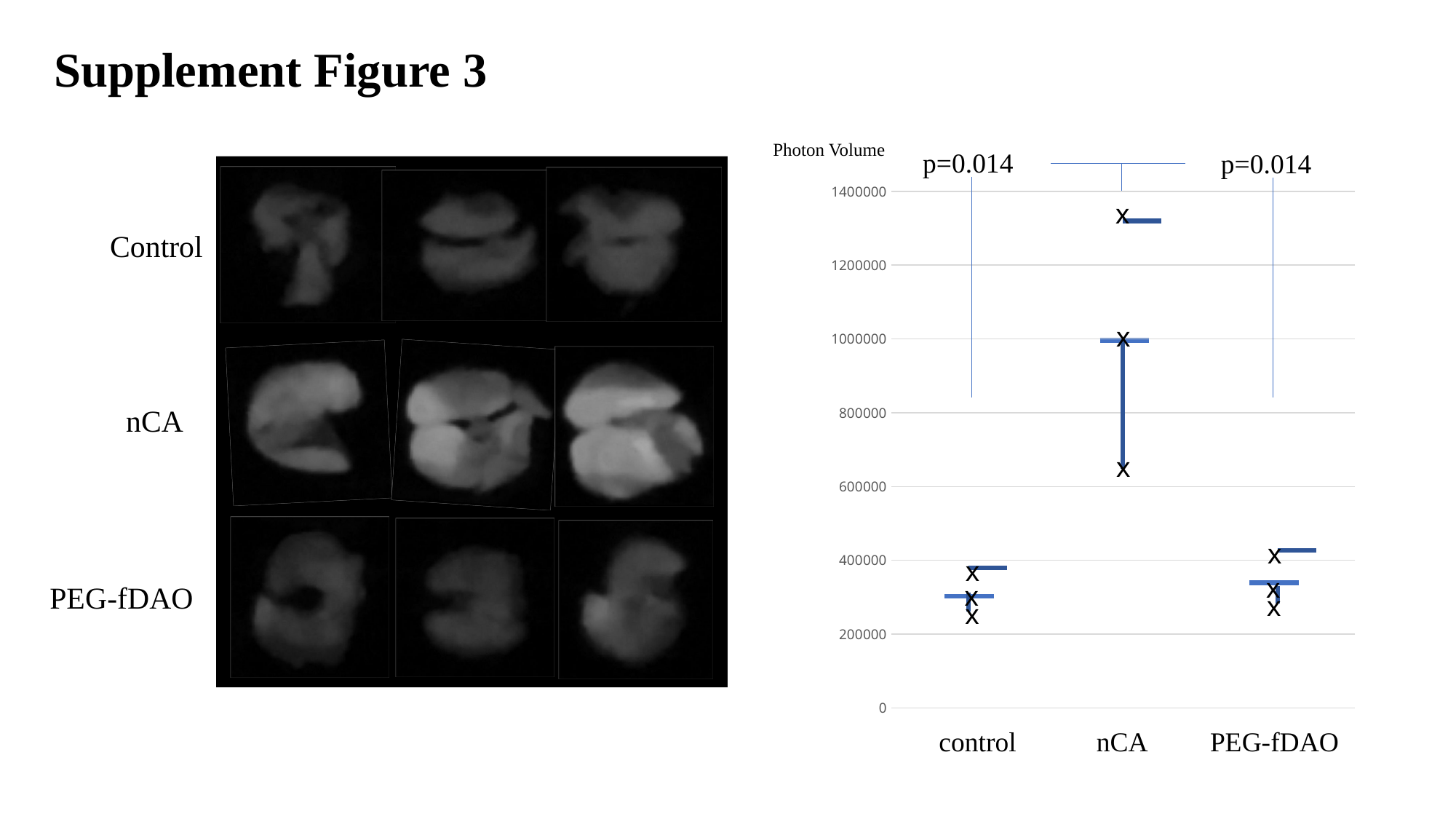

Supplement Figure 3
Photon Volume
p=0.014
p=0.014
[unsupported chart]
x
Control
x
nCA
x
x
x
x
PEG-fDAO
x
x
x
control
nCA
PEG-fDAO

## Slide 5
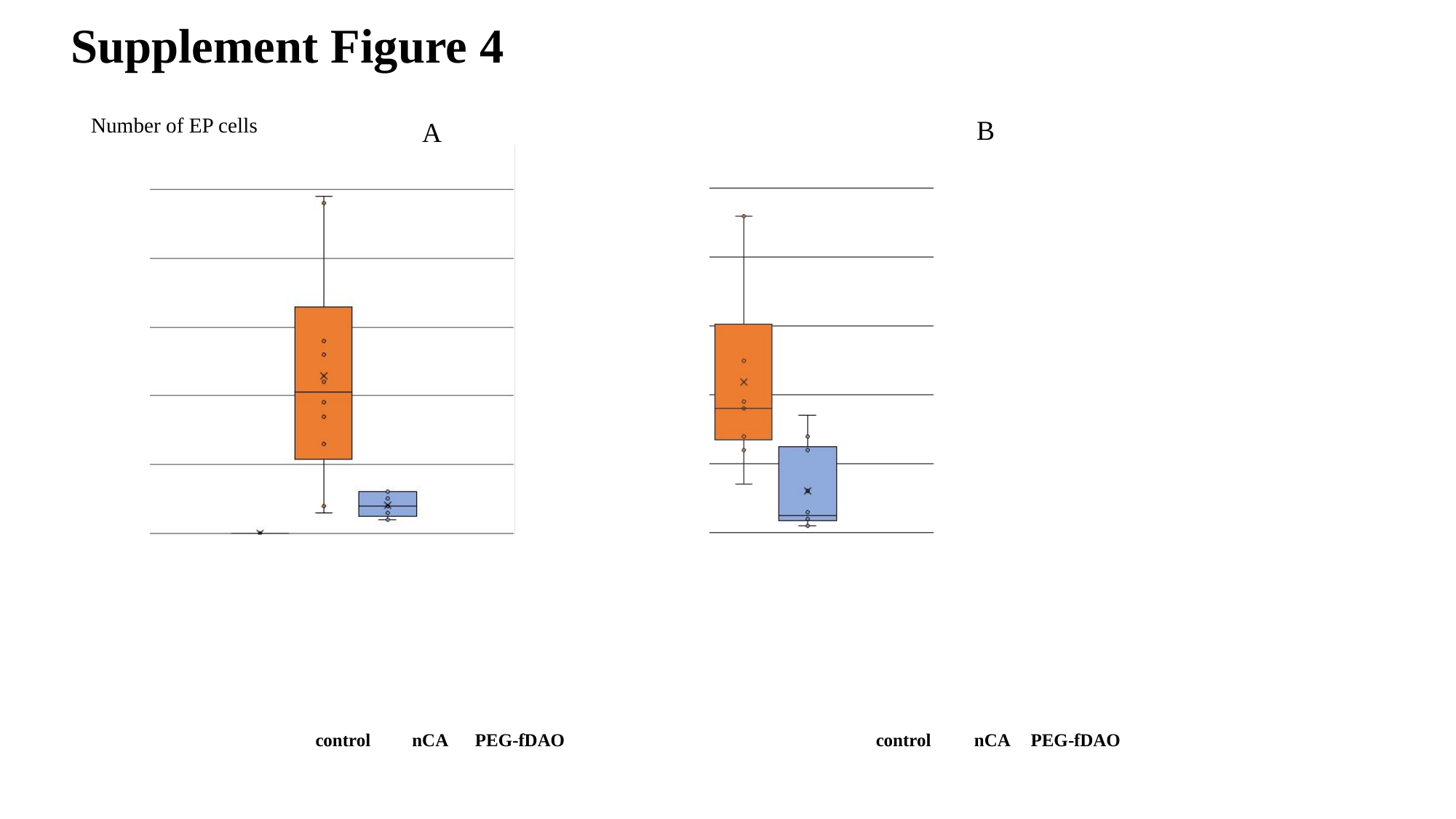

Supplement Figure 4
Number of EP cells
B
A
control
nCA
PEG-fDAO
control
nCA
PEG-fDAO
