# Supplementary material for: Treatment with Polyethylene Glycol–Conjugated Fungal d-Amino Acid Oxidase Reduces Lung Inflammation in a Mouse Model of Chronic Granulomatous Disease
Source: Inflammation. 2022 Feb 24;45(4):1668–79. doi: 10.1007/s10753-022-01650-z (PMC9197883; doi:10.1007/s10753-022-01650-z)
Supplement: Supplementary file 2 — Supplementary file2 (PPTX 8416 KB) [file 10753_2022_1650_MOESM2_ESM.pptx]

## Slide 1
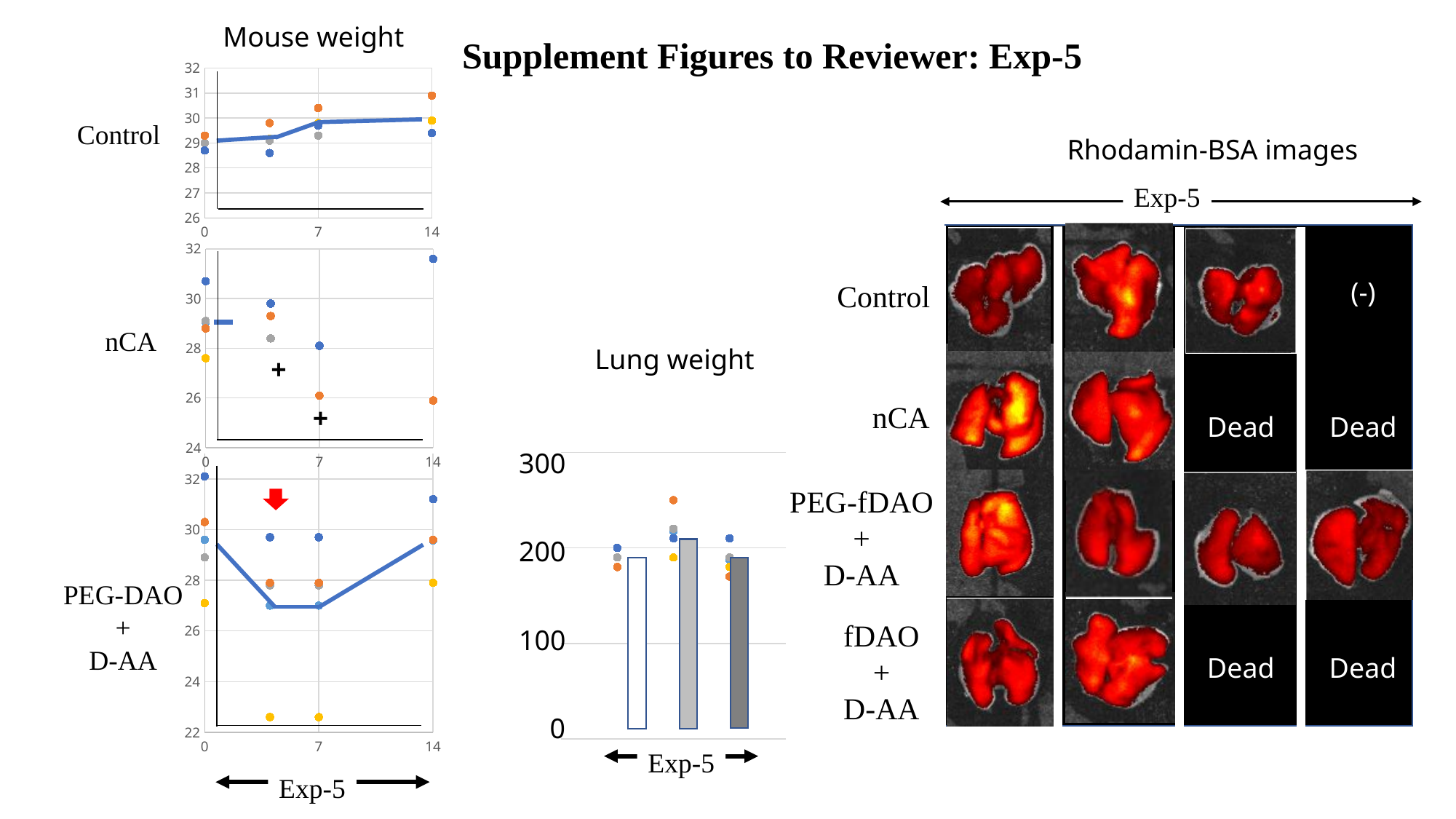

Mouse weight
Supplement Figures to Reviewer: Exp-5
### Chart
| Category | Control | | | |
|---|---|---|---|---|Control
Rhodamin-BSA images
Exp-5
### Chart
| Category | CA | | | | |
|---|---|---|---|---|---|(-)
Control
nCA
Lung weight
+
nCA
+
Dead
Dead
300
### Chart
| Category | 1 | 2 | 3 | 4 | |
|---|---|---|---|---|---|
### Chart
| Category | | | | | |
|---|---|---|---|---|---|
PEG-fDAO
+
D-AA
200
PEG-DAO
+
D-AA
fDAO
+
D-AA
100
Dead
Dead
0
Exp-5
Exp-5

## Slide 2
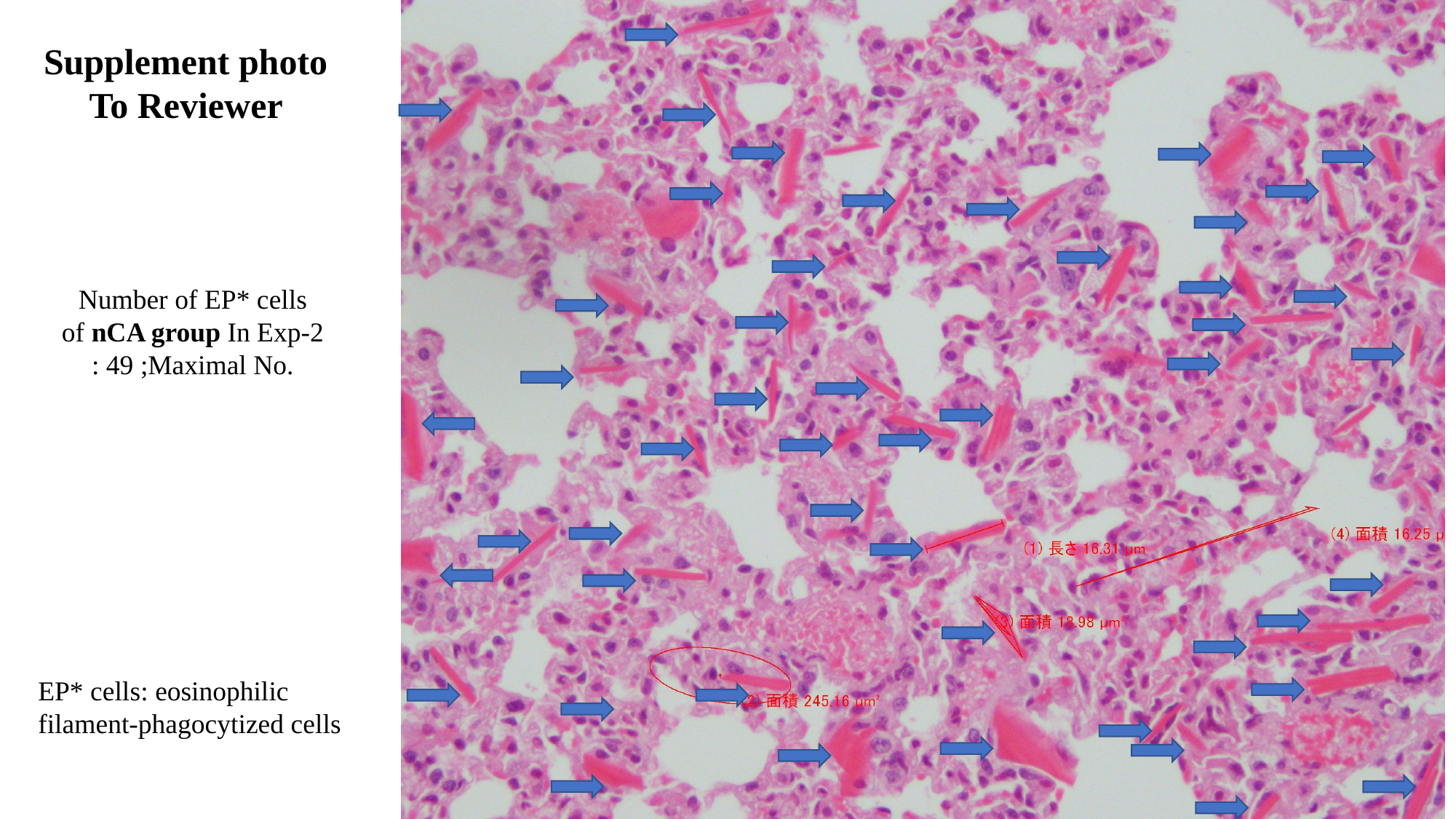

Supplement photo
To Reviewer
Number of EP* cells
of nCA group In Exp-2
: 49 ;Maximal No.
EP* cells: eosinophilic
filament-phagocytized cells

## Slide 3
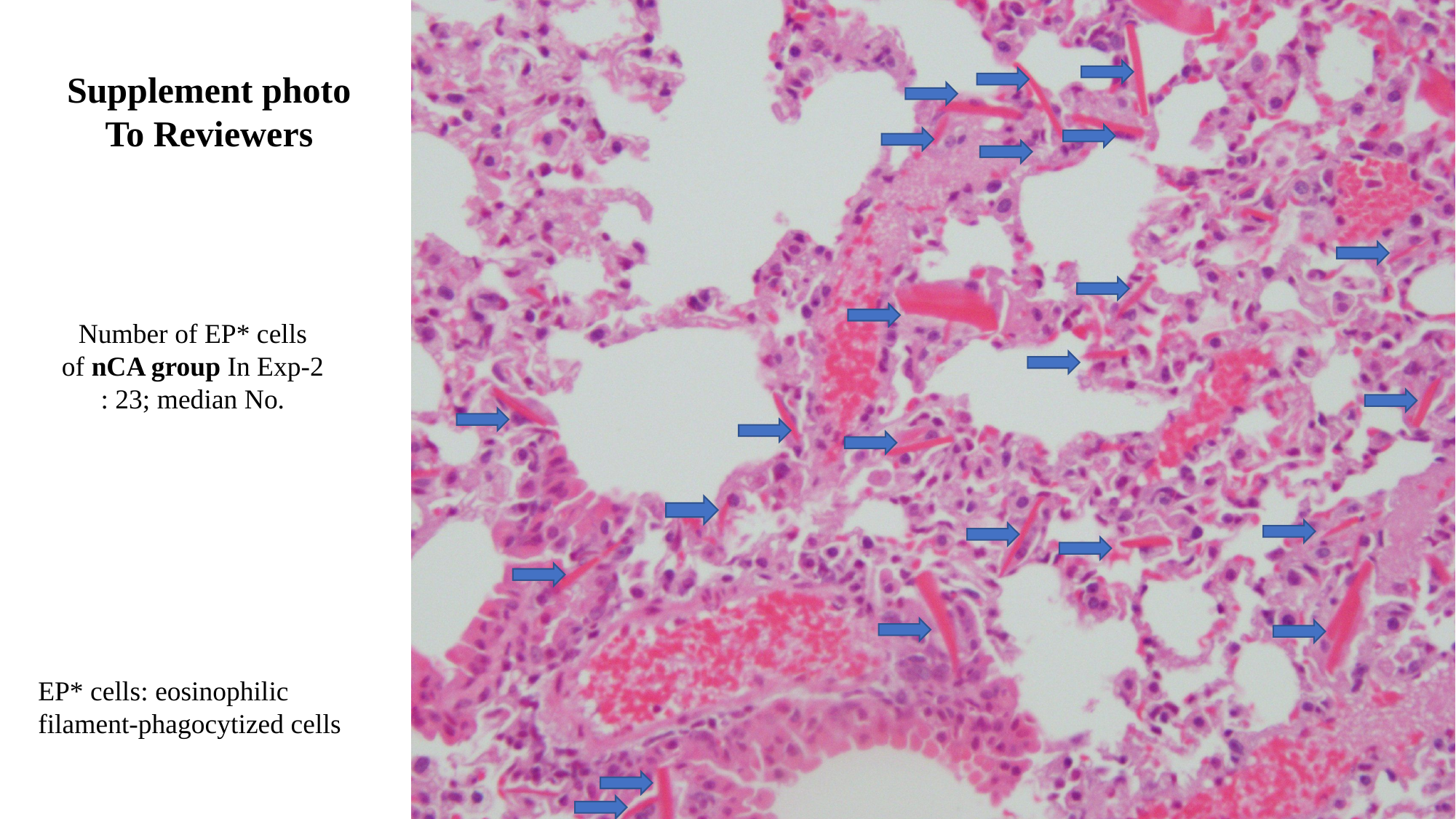

Supplement photo
To Reviewers
Number of EP* cells
of nCA group In Exp-2
: 23; median No.
EP* cells: eosinophilic
filament-phagocytized cells

## Slide 4
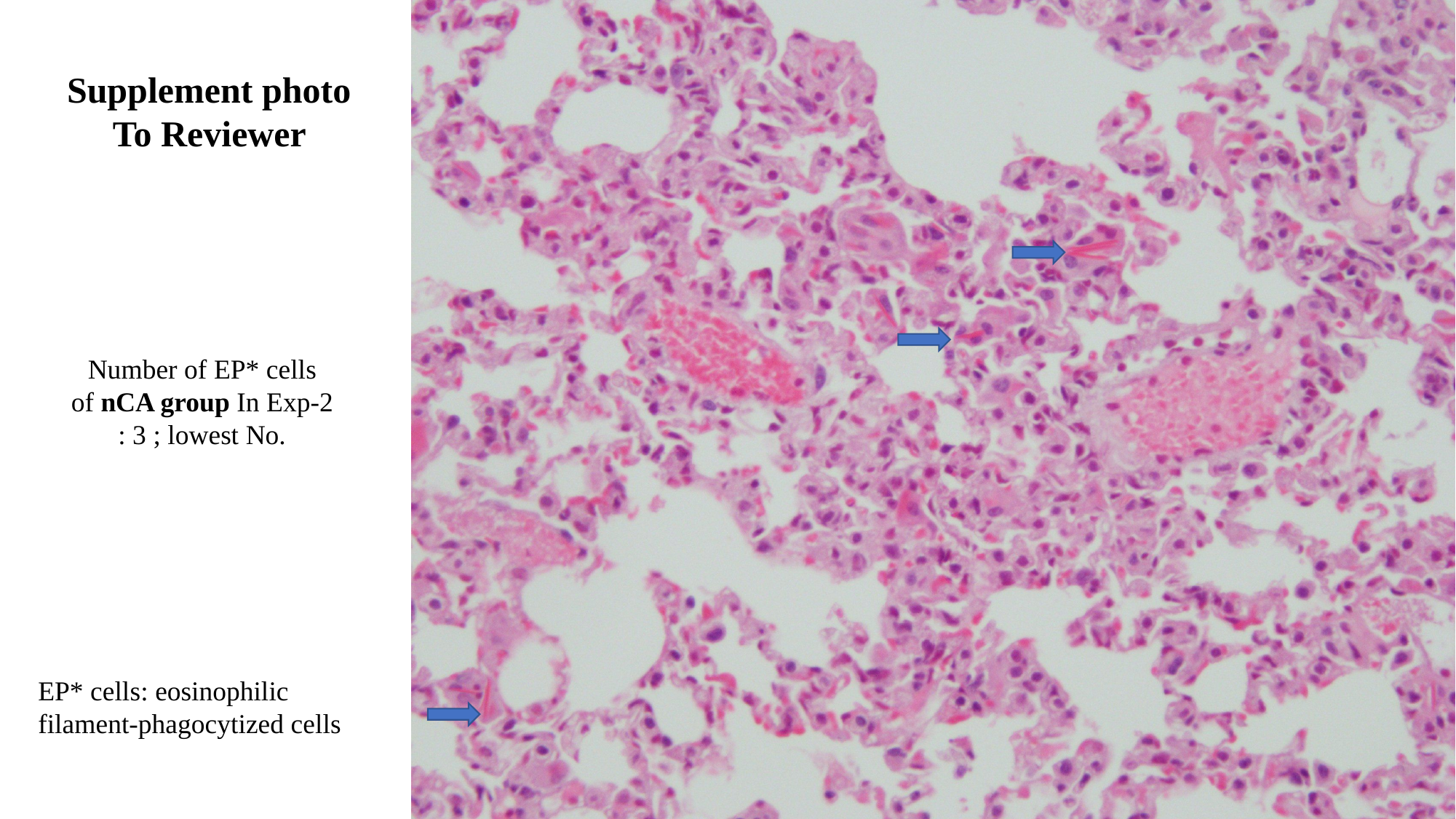

Supplement photo
To Reviewer
Number of EP* cells
of nCA group In Exp-2
: 3 ; lowest No.
EP* cells: eosinophilic
filament-phagocytized cells

## Slide 5
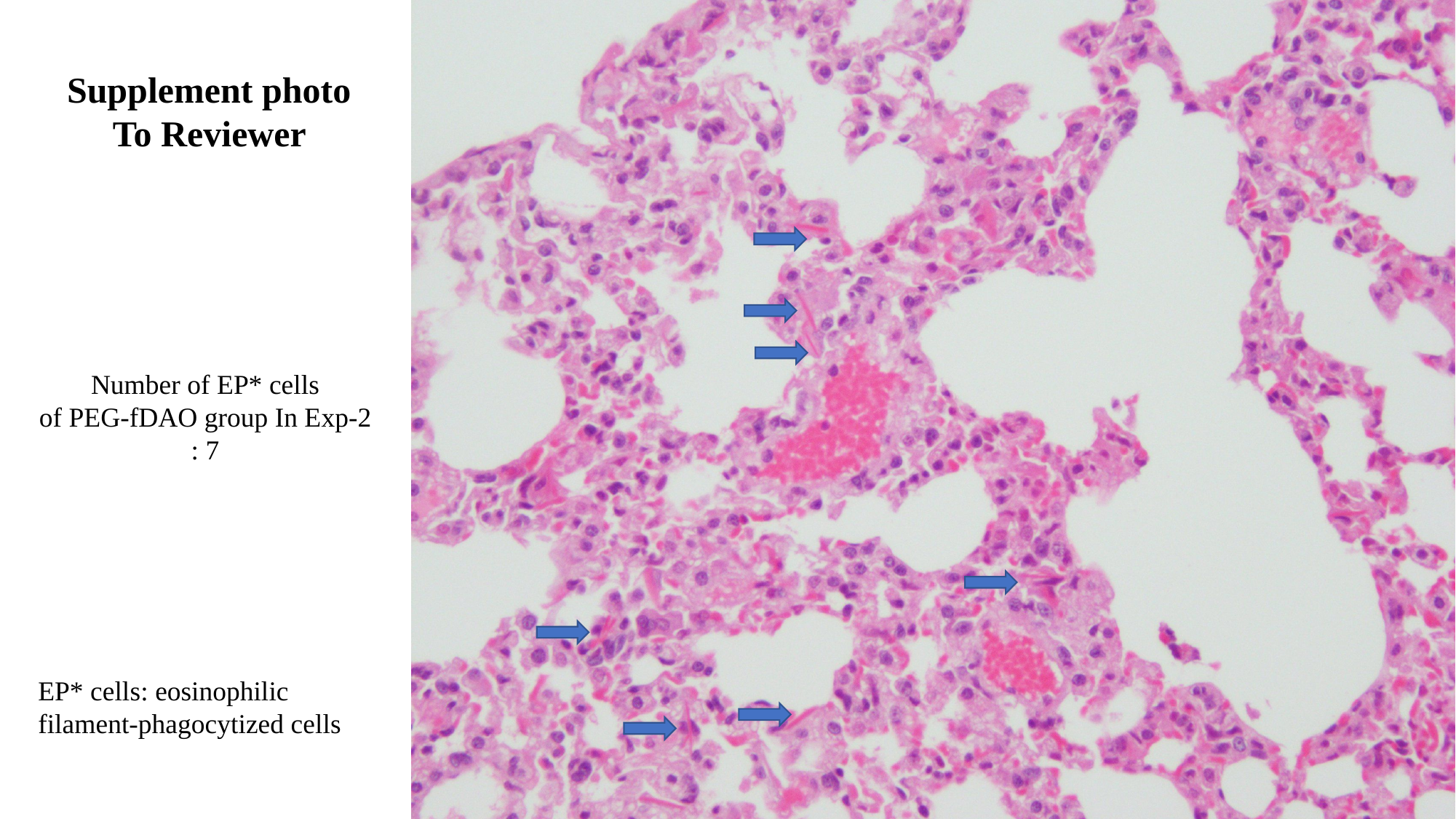

Supplement photo
To Reviewer
Number of EP* cells
of PEG-fDAO group In Exp-2
: 7
EP* cells: eosinophilic
filament-phagocytized cells

## Slide 6
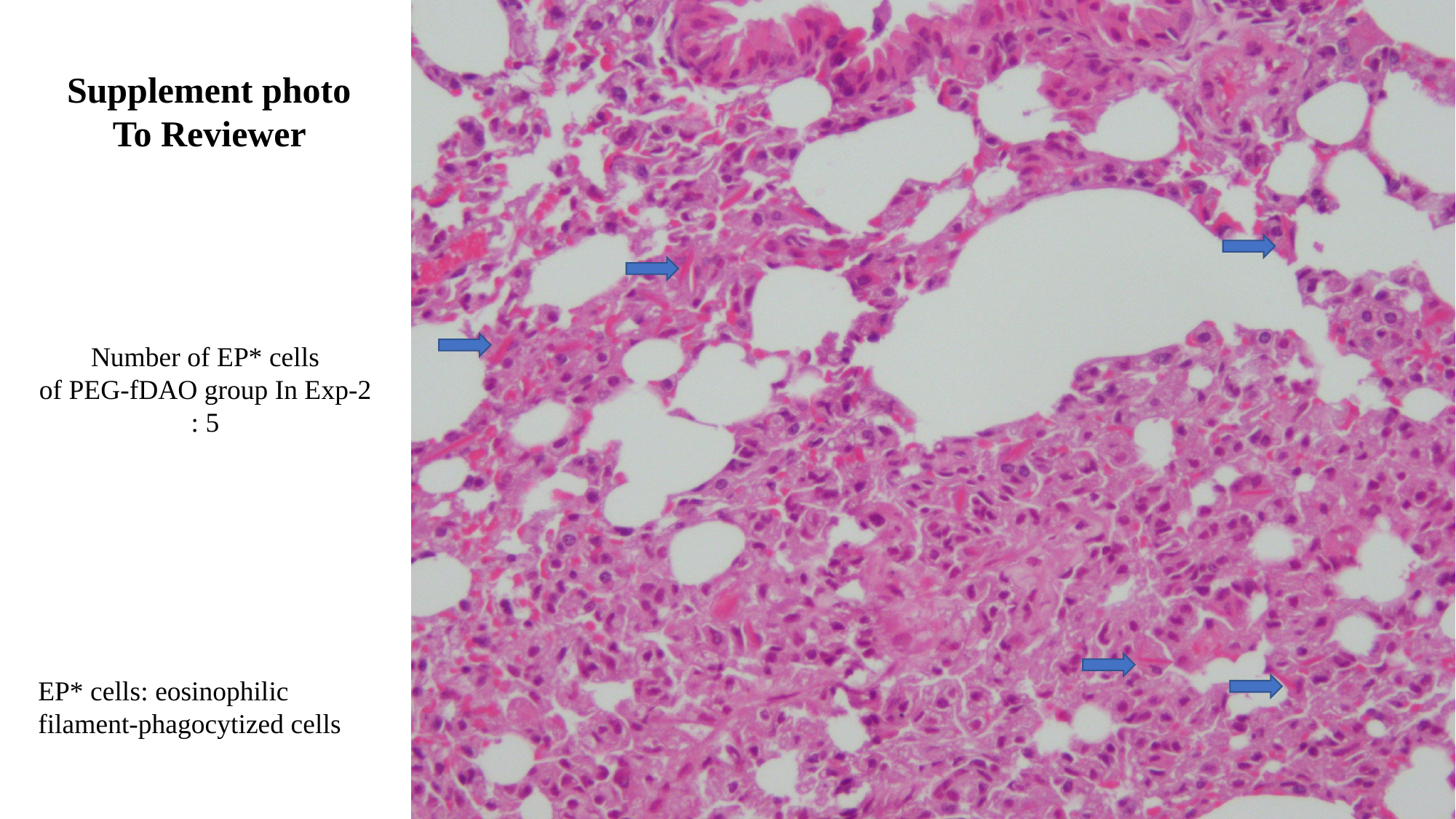

Supplement photo
To Reviewer
Number of EP* cells
of PEG-fDAO group In Exp-2
: 5
EP* cells: eosinophilic
filament-phagocytized cells

## Slide 7
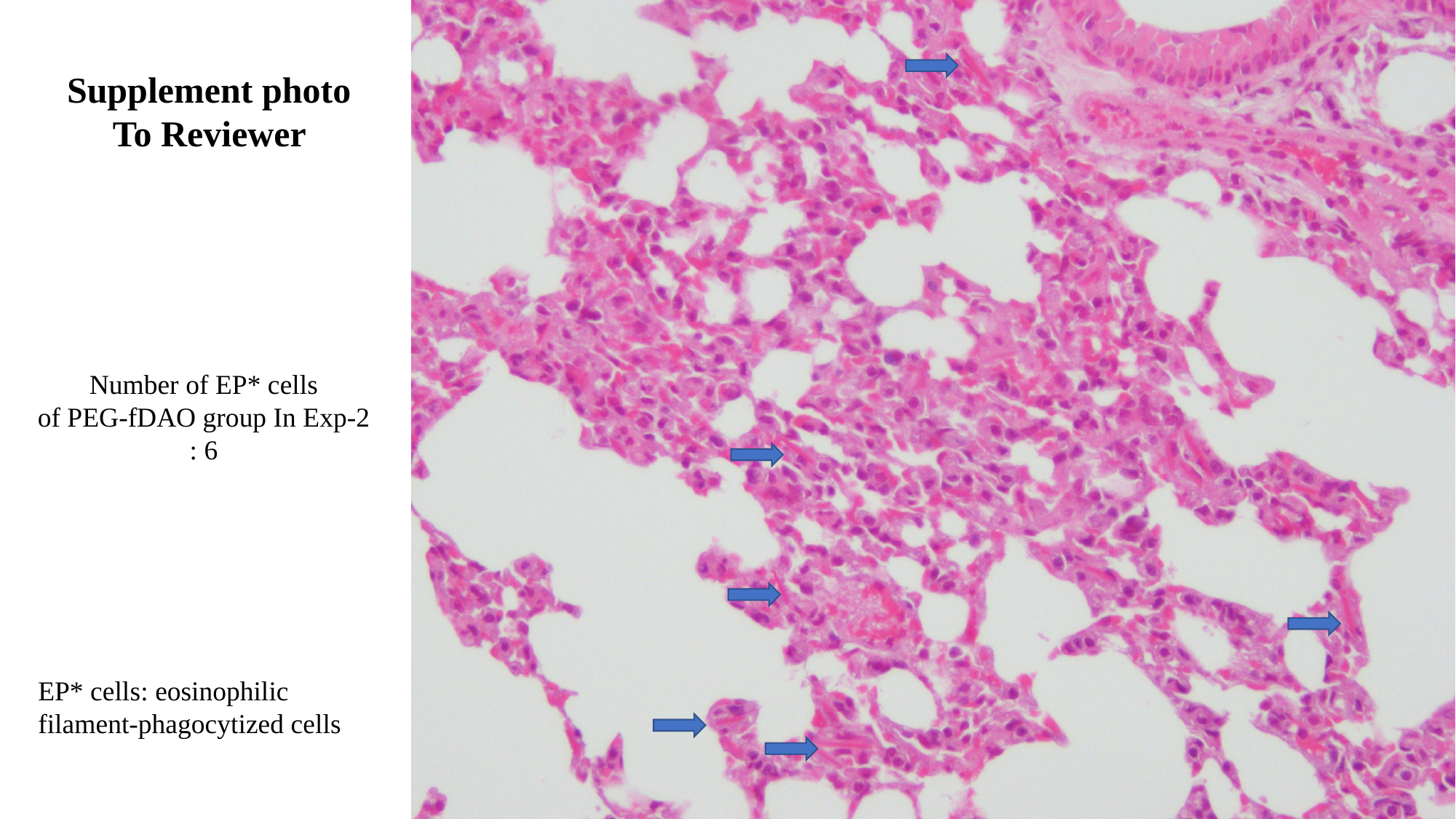

Supplement photo
To Reviewer
Number of EP* cells
of PEG-fDAO group In Exp-2
: 6
EP* cells: eosinophilic
filament-phagocytized cells

## Slide 8
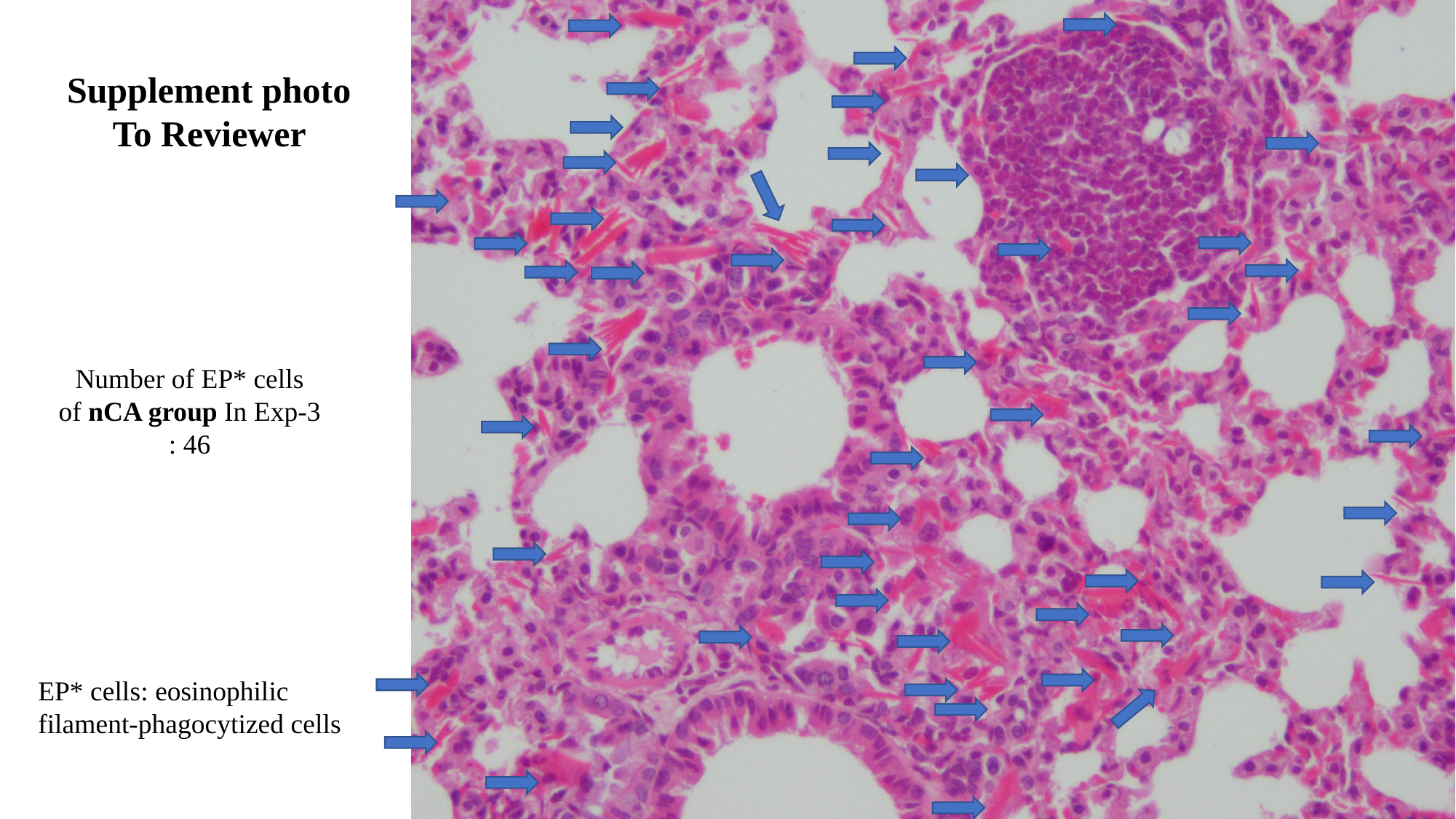

Supplement photo
To Reviewer
Number of EP* cells
of nCA group In Exp-3
: 46
EP* cells: eosinophilic
filament-phagocytized cells

## Slide 9
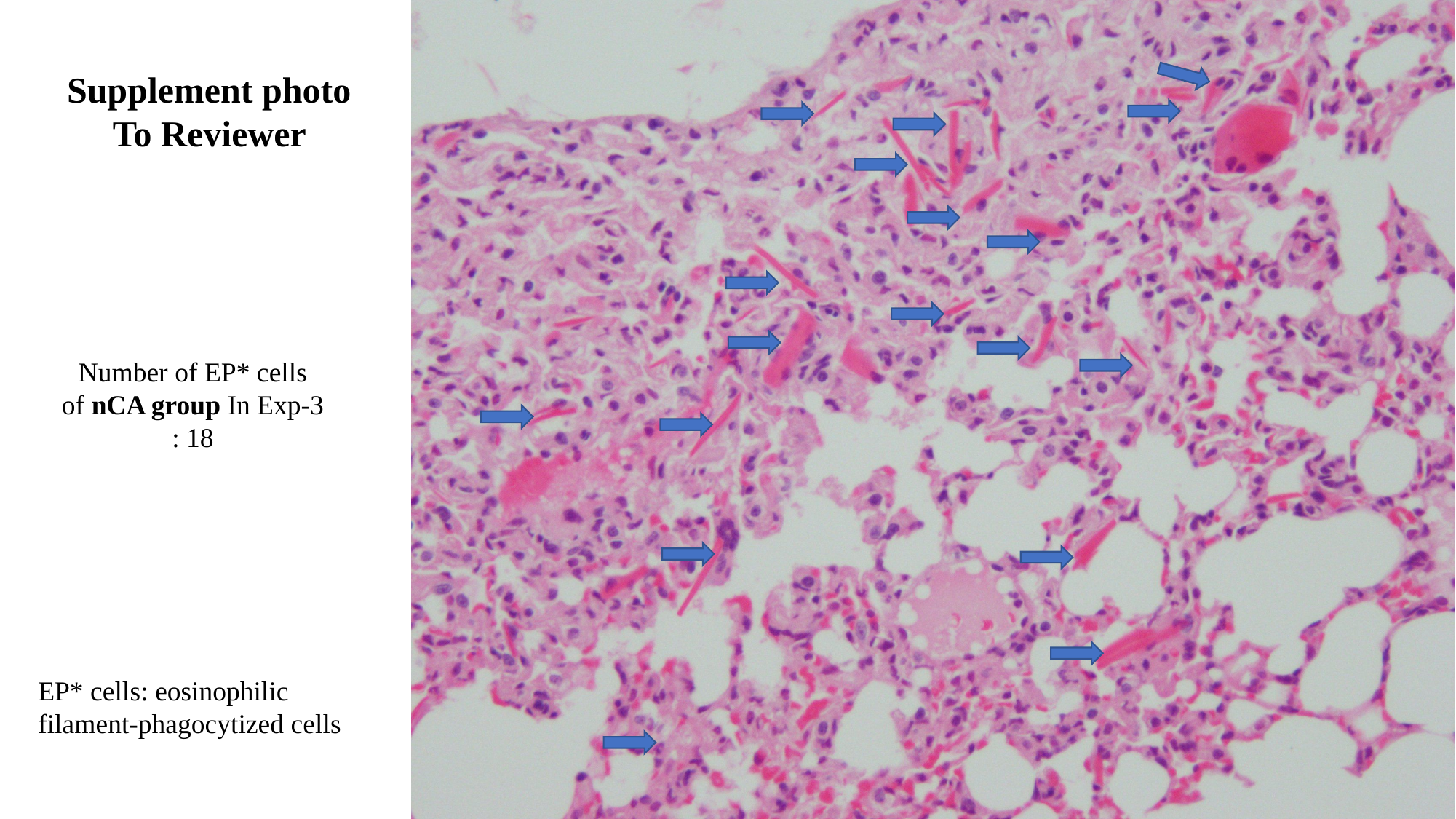

Supplement photo
To Reviewer
Number of EP* cells
of nCA group In Exp-3
: 18
EP* cells: eosinophilic
filament-phagocytized cells

## Slide 10
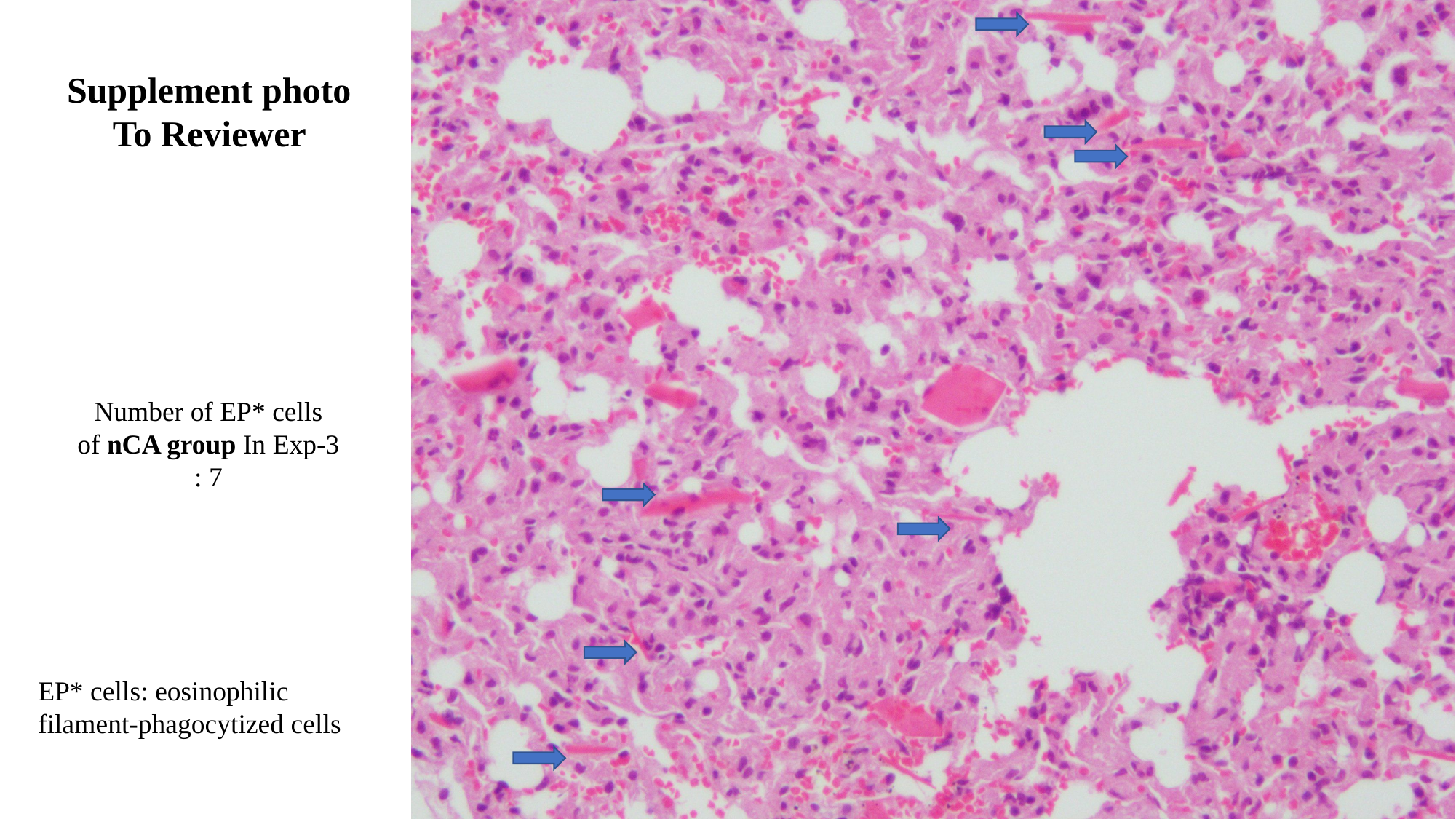

Supplement photo
To Reviewer
Number of EP* cells
of nCA group In Exp-3
: 7
EP* cells: eosinophilic
filament-phagocytized cells

## Slide 11
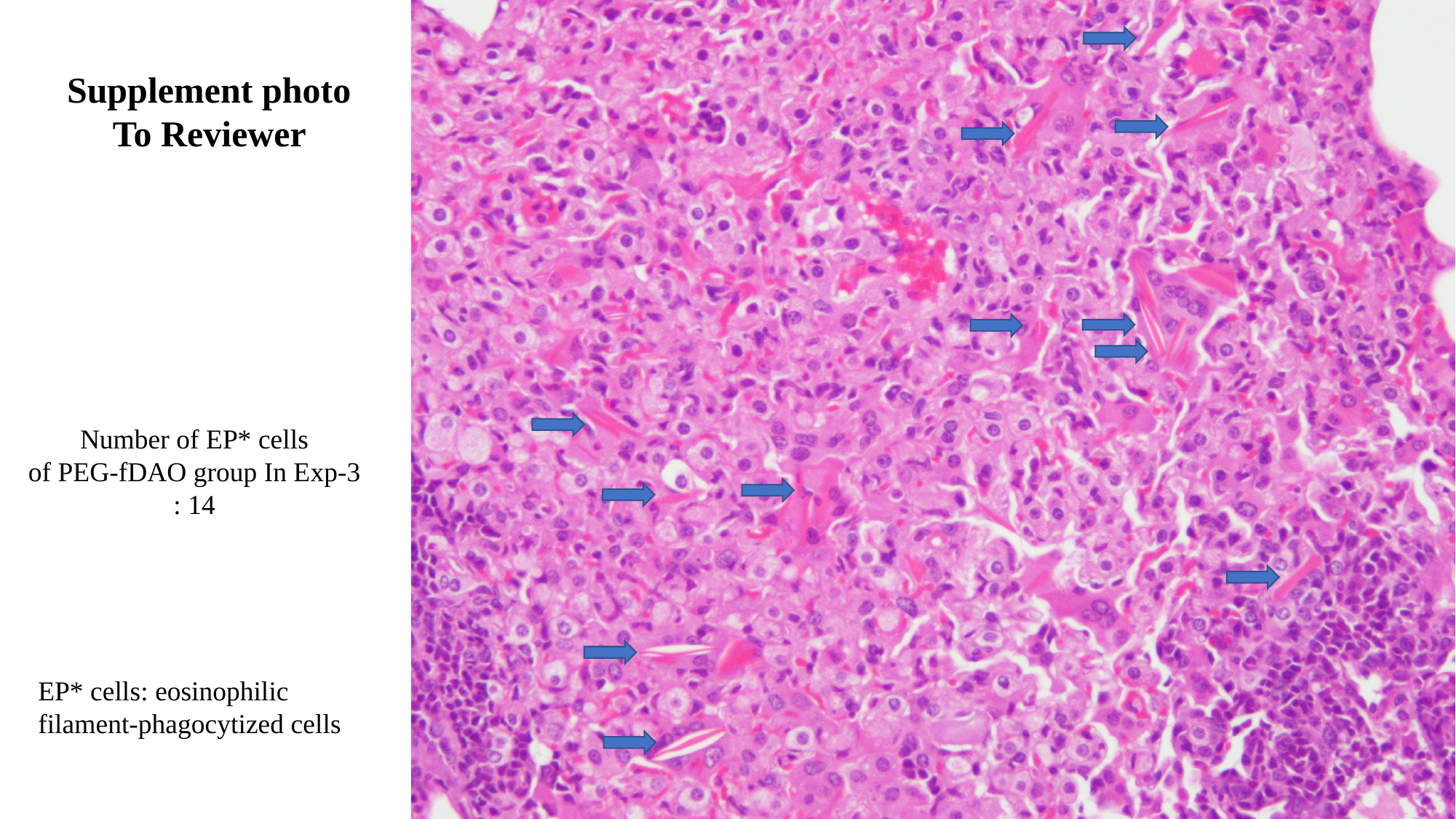

Supplement photo
To Reviewer
Number of EP* cells
of PEG-fDAO group In Exp-3
: 14
EP* cells: eosinophilic
filament-phagocytized cells

## Slide 12
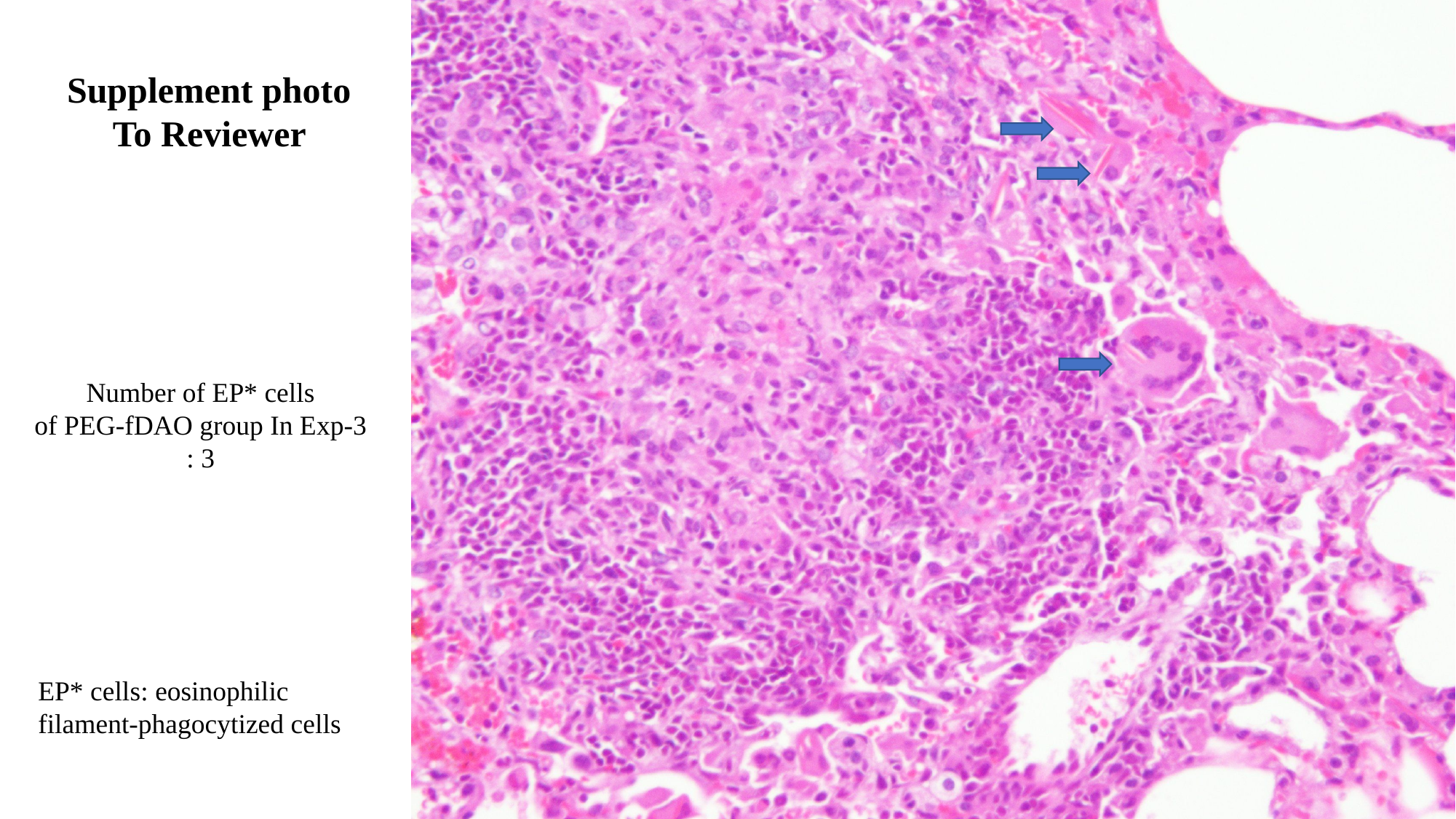

Supplement photo
To Reviewer
Number of EP* cells
of PEG-fDAO group In Exp-3
: 3
EP* cells: eosinophilic
filament-phagocytized cells

## Slide 13
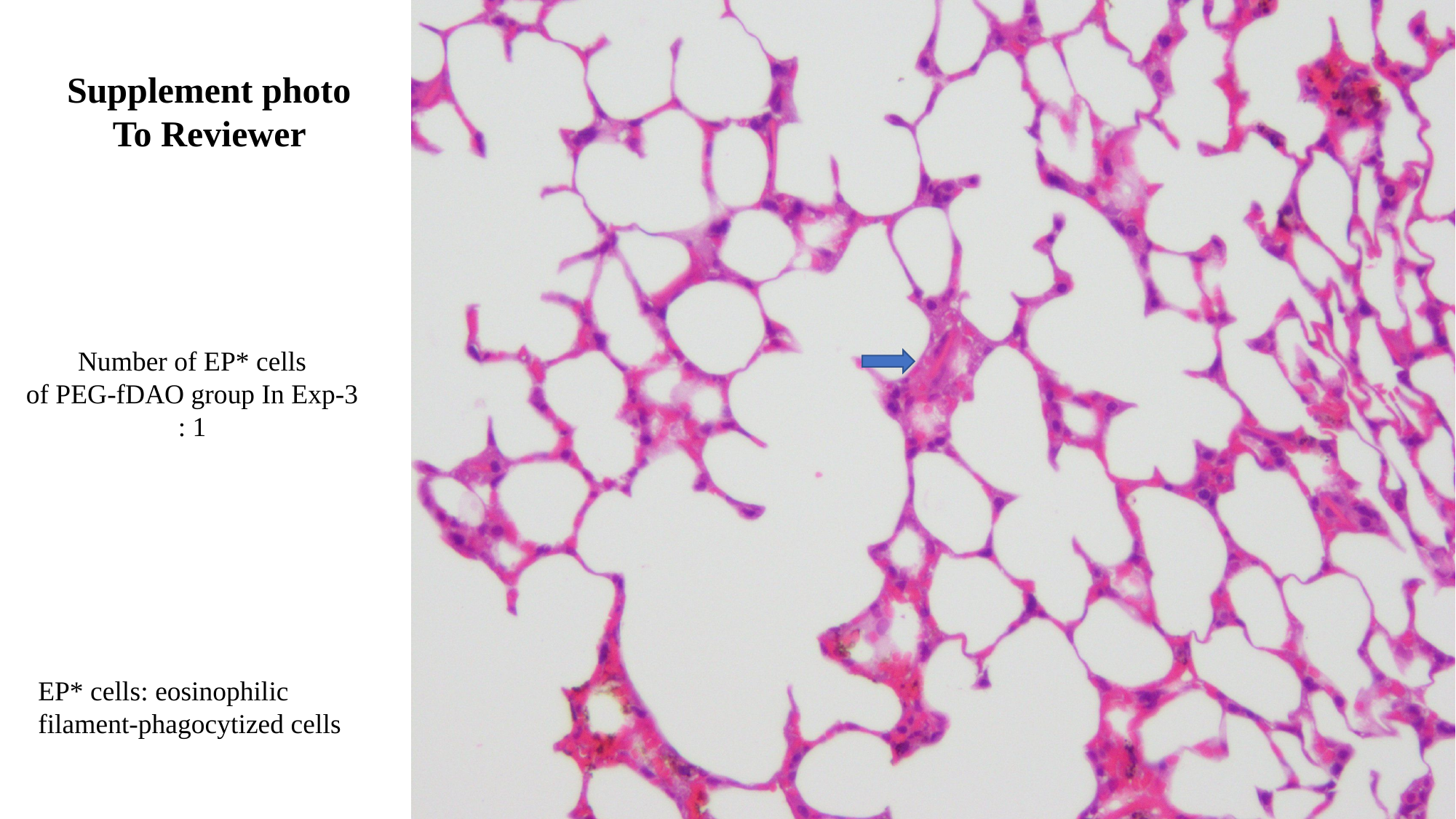

Supplement photo
To Reviewer
Number of EP* cells
of PEG-fDAO group In Exp-3
: 1
EP* cells: eosinophilic
filament-phagocytized cells
